# Evaluation of host-immune biomarker signatures as multiplex qPCR diagnostic assays: a pilot study toward meeting WHO target product profiles for TB diagnosis in India

**DOI:** 10.3389/ftubr.2025.1678039

**Published:** 2026-02-16

**Authors:** Harriet N. Garlant, Kalaiarasan Ellappan, Noyal Mariya Joseph, Carrie Turner, Vishnukanth Govindaraj, Saka Vinod Kumar, Sanjeev Kumar, Seshadri Vasan, Karen E. Kempsell

**Affiliations:** 1 UK Health Security Agency, Porton Down, Salisbury, United Kingdom; 2 Department of Microbiology, Jawaharlal Institute of Postgraduate Medical Education and Research, Puducherry, India; 3 Department of Pulmonary Medicine, Jawaharlal Institute of Postgraduate Medical Education and Research, Puducherry, India; 4 School of Medical and Health Sciences, Edith Cowan University, Joondalup, WA, Australia

**Keywords:** tuberculosis, biomarker, immune, diagnosis, qPCR

## Abstract

**Background:**

Tuberculosis (TB) remains the leading cause of death from a single infectious agent. Current diagnostic tools are limited, especially in low-resource settings. The World Health Organization's (WHO) Target Product Profiles (TPPs) call for rapid, non-sputum-based diagnostics with high sensitivity and specificity. This study evaluates our previously published TB-associated host-immune biomarkers alongside small-size signatures from other studies, in our previously published non-human primate (NHP) TB infection study dataset (GSE76703) and two previously published human datasets (GSE144127, GSE42834), which include other disease group comparators, including sarcoidosis. These were also evaluated in a small-scale, exploratory qPCR pilot study to assess the feasibility of implementing these previously validated signatures in a South Indian TB patient cohort, comparing their diagnostic performance against WHO TPP criteria.

**Methods:**

Twenty-six genes from published signatures (INDUK, Roe1/Roe3, Sweeney3, RISK6) were analyzed in these NHP and human datasets, using network and machine learning approaches, prior to exploratory evaluation using single and multiplex qPCR assays. These were tested using peripheral blood sample RNAs from pulmonary TB (PTB) (*n* = 15) and extrapulmonary TB (EPTB) (*n* = 15) patients and high-incidence controls (*n* = 15). The diagnostic performance of biomarkers, prior signatures, and novel promising combinations were assessed against WHO TPPs for triage and confirmatory tests.

**Results:**

Several biomarker signatures successfully distinguished active TB ((ATB) PTB and EPTB combined) from controls. The minimal INDUK signature (*GBP1* + *IFIT3*) met the optimal TPP criteria for both triage and confirmatory testing for PTB (100% sensitivity and specificity, area under the receiver operating characteristic curve (AUROC:1)) and achieved the 80% sensitivity, 100% specificity threshold for EPTB (AUROC: 0.92 CI: 0.8261–1.00). Combined signatures incorporating genes from INDUK, Roe1, and Sweeney3 further improved diagnostic accuracy for ATB overall (AUROC: 0.98 95% CI: 0.9472–1.00).

**Conclusion:**

This preliminary pilot study demonstrates successful evaluation of biomarker signatures as diagnostic qPCR assays for TB diagnosis and, to our knowledge, is the first study to demonstrate the potential for combined host-immune biomarker signatures from different studies that meet WHO TPP benchmarks. These findings support the potential for the development of low-cost, field-adaptable diagnostic tools. Further validation is now under way on a larger cohort of TB patients and controls.

## 1 Introduction

Despite being a treatable disease, tuberculosis (TB) continues to impose a significant global health burden. Since 2020, TB incidence has risen by 6.48%, largely attributed to pandemic-related disruptions and persistent diagnostic gaps, suggesting a growing population of undiagnosed individuals who may continue to transmit the disease. These developments represent a significant setback to the World Health Organization's (WHO) End TB Strategy, which aims to eliminate TB as a public health threat by 2030 through enhanced prevention, diagnosis, and treatment (1). According to the WHO Global Tuberculosis Report, only 48% of individuals with active TB were diagnosed using rapid molecular testing for TB in 2023, underscoring the persistent diagnostic gap (2–5). Addressing this diagnostic inadequacy remains critical. Insufficient access to fast, high-quality, affordable TB diagnostics and inadequate linkage to care continue to hinder global control efforts (6). There is an urgent need for scalable, cost-effective diagnostic tools to ensure timely detection and treatment, an essential step toward achieving the End TB milestones.

In 2014, the WHO published Target Product Profiles (TPPs) to guide the development of new TB diagnostic tests, delineating their expected performance requirements, with an updated revision in 2024 (7–9). These TPPs, outlining minimum and optimal test criteria for diagnosing active TB (ATB), included (i) a point-of-care (POC) biomarker-based, non-sputum test; (ii) a POC triage test to identify individuals requiring further evaluation; and, in 2024, (iii) an updated TPP requirement for a rapid TB detection test for community-based testing with definitions for classes of POC tests, near-POC tests, and low-complexity tests (9). The minimum and optimal requirements for each TPP vary according to clinical manifestation. Extrapulmonary TB (EPTB), which involves anatomical sites beyond the lungs, is estimated to account for over 20% of TB cases in India and other high-burden countries (10–12). Conventional diagnostic tools, such as sputum microscopy and chest radiography, remain challenging for PTB and are largely ineffective for EPTB due to its heterogeneous clinical presentations, necessitating reduced TPP requirements set for EPTB. For the confirmatory test, optimal requirements were set at >98% sensitivity for smear-positive, culture-positive pulmonary TB (PTB), >68% for smear-negative, culture-positive PTB, and >80–85% for EPTB. Minimum requirements were set at >65% sensitivity and 98% specificity for both PTB and EPTB to ensure performance comparable to GeneXpert MTB/RIF assays. For a triage test, optimal requirements were set at 95% sensitivity and 80% specificity. Minimum requirements were selected as 90% sensitivity and 70% specificity. The 2025 update for rapid TB detection tests reports reduced sensitivity requirements for non-sputum-based specimens, with an optimal sensitivity threshold of >95% and minimal sensitivity thresholds of 80%, 75%, and 65% for low complexity, near point-of-care (POC), and POC tests, respectively (9). Specificity remains at >98%. These thresholds serve as critical standards for evaluating the clinical utility of emerging diagnostic tools. To be considered viable for implementation, any new test must meet at least the minimum TPP criteria and ideally approach optimal performance standards.

India bears one of the highest TB burdens globally, accounting for approximately one-fifth of the total global TB incidence and with 31–41% of the population estimated to be infected (10). Currently, key reference standards for TB diagnosis in India are dependent on whether the ATB presentation is PTB or EPTB, what resources are available, and whether drug resistance is being assessed. According to the India TB report 2022, Mycobacteria Growth Indicator Tube (MGIT) remains among the most sensitive TB detection methods, yet much attention has been focused on upgrading diagnostic capacities by introducing precision and molecular diagnostics (12). Affordability, scalability, and ease of integration into existing health systems are also important considerations to ensure widespread access and impact, particularly in high-burden, low-income countries (2, 13, 14).

Blood-based assays using disease-associated biomarkers have shown promise for diagnosing various conditions, including TB (15). While biomarkers span multiple biological types (16), much research has focused on differentially expressed RNAs, such as mRNA, miRNAs, and lncRNAs, for TB profiling. Host transcriptional signatures from peripheral blood can indicate disease status, predict outcomes (17–21), identify at-risk latently infected individuals (20, 22–24), and monitor treatment response (4, 20, 25–29). These findings support the development of specific, scalable biomarkers for cost-effective screening (30). Numerous signatures now distinguish ATB from healthy individuals with comparable area under the receiver operating characteristic curve (AUROC) performance (3, 20, 28, 31–45). Several of these host biomarker signatures for ATB have undergone comparative evaluation, with many validated across diverse datasets (18, 20, 23, 30, 46–49). In a South African cohort, Turner *et al*. evaluated a number of panels, and of these, four top-performing panels were identified: Sweeney3 ([*GBP5*+*DUSP3*]/2)-*KLF2*, Roe1 (*BATF2*), Roe3 (*BATF2*+*GBP5*+*SCARF1*), and Kaforou25, each demonstrating high diagnostic accuracy (>86%) regardless of age, sex, HIV status, prior TB infection, or smear result (18). Roe et al. showed that *BATF2* alone could distinguish ATB from healthy individuals (AUROCs: 0.98 and 0.96), while a separate four-gene signature was needed to differentiate ATB from pneumonia (34). Though most studies focused on PTB, Mann *et al*. found Roe3 performed best in presumptive EPTB cases (AUROC: 0.77–0.82) (48). In separate studies, the RISK6 signature, developed as a PCR-based tool for screening and treatment monitoring, has shown consistent performance across settings (19, 28, 37, 50). Warsinke et al. reported that only 2 of 16 tested signatures met WHO's minimum TPP criteria (51).

Many of these biomarker signatures exhibit overlapping profiles, e.g., *GBP5* in the Roe3 and Sweeney3 panels. *GBP1* is identified as a key candidate in many studies (24, 40, 44, 45, 52–54) along with *GBP5* and other key genes, e.g., *GBP2, BATF2, IFIT3, IFITM3*. These are predominantly interferon-inducible genes, components of the innate immune response to viral and intracellular pathogens (27, 35, 37, 51) and are significantly upregulated in TB (32, 54, 55), in addition to those with other immune functions, e.g., *SAMD9L* (52). Various combinations of these genes, along with other genes from the same family, e.g., *GBP3-GBP6*, are all represented as major components of biomarker signatures from other groups (54). It is becoming clear that many of these signatures demonstrate similar performance, perhaps due to the overlapping functionality of key biological processes shared between gene targets. Some are already in development/clinical evaluation on commercially available diagnostic platforms (2, 13, 14), i.e., Sweeney3 (4, 56–65) and RISK6.^1^ However, none have yet met the WHO optimum requirements for a confirmatory test. Work is required to improve the performance of TB-associated immune biomarker panels to meet these WHO TPP requirements.

We previously identified biomarker signatures of PTB in a *Macaca fascicularis* non-human-primate (NHPP) model of PTB, with the expression of many biomarkers such as *GBP1* overlapping with human datasets (55). Further validation as both mRNA and protein targets in the United Kingdom (UK) and India cohorts has demonstrated diagnostic capability (54, 66). Various combinations of these biomarkers effectively discriminated PTB and EPTB from healthy controls. A preferred set of biomarkers distinguished patients with ATB, i.e., *GBP1, CALCOCO2, IFIT3, IFITM3, SAMD9L*, and *SNX10*, of which a refined combination signature of *GBP1, IFIT3*, and *SAMD9L* was able to meet WHO TPP specifications (54, 66). In this study, we further evaluate these six biomarkers in comparison with the Roe1 (34), Roe3 (67), Sweeney3 (36), and RISK6 (28, 50) signatures, utilizing analysis of published datasets and development and validation of qPCR assays to define targets and combinations that meet TPP requirements. The aim is to develop assays for use on widely accessible, qPCR platforms, routinely used in diagnostic laboratories and proven effective for biomarker assay implementation (33). Assays were configured in duplex and quadruplex formats and tested in an exploratory, pilot cohort study of PTB and EPTB patients alongside high-incidence region controls (CNTRL) from a South Indian tertiary hospital. Our assays successfully discriminated PTB and EPTB patients from CNTRL, demonstrating POC for qPCR-based diagnostics for ATB. Furthermore, random forest analysis, an established high-accuracy method for TB biomarker selection (68), identified novel biomarker combinations with superior performance. The preliminary results indicate that these can meet WHO's optimal TPP criteria for confirmatory testing; however, upscaled testing in larger cohort studies is required.

## 2 Materials and methods

### 2.1 STRING functional enrichment analysis of UK Health Security Agency (UKHSA), Roe1/3, Sweeney3, and RISK6 biomarkers and GeneSpring analysis using previously published datasets

#### 2.1.1 STRING version 12.0 functional enrichment analysis

Protein–protein interaction (PPI) networks for all combined UKHSA, Roe1/3, Sweeney3, and RISK6 biomarkers were constructed using the STRING database (Search Tool for the Retrieval of Interacting Genes/Proteins; version 12.0 (69–71, 115)) to characterize host immune-related protein interactions. This algorithm provides statistical and functional enrichment information based on various database sources, including Biological Process (Gene Ontology), GO terms, Reactome pathways, and reference publications (PubMed), among others. Outputs delineate protein interactions greater than those that would be expected by chance for a random set of proteins of the same size and degree distribution drawn from the genome, indicating proteins that are at least partially biologically connected as a group. Study data values were not included as part of this analysis. In short, a complete list of study gene identifiers was uploaded to the STRING analysis tool portal using the Multiple Proteins by Names/Identifiers setting and analysis initiated using search tool default settings, using *Homo sapiens* as the reference organism. Interactions supported by experimental evidence, curated databases, co-expression, or text mining with scores of medium confidence = 0.4, false discovery rate (FDR) ≤ 0.05, minimum signal ≥0.01, minimum strength ≥0.01, minimum count in network = 2 were included. Clustered networks were visualized using the integrated STRING network viewer to highlight interaction density, cluster boundaries, and hub proteins. Network images and other outputs were downloaded from the portal using various functions in the export tool.

Functionally related groups of immune proteins were further delineated using K-means clustering. Each protein (node) was represented as a vector in a multidimensional interaction space. The algorithm partitioned nodes into K clusters by minimizing the within-cluster sum of squared distances between each node and its cluster centroid. Centroids and memberships were updated iteratively until convergence, generating stable clusters of proteins with similar interaction profiles. The number of clusters (K) was set manually to K = 7. Each cluster was interpreted as a putative functional module, representing host proteins potentially involved in coordinated immune signaling or defense pathways during *M. tuberculosis* infection.

#### 2.1.2 GeneSpring version 14.9 analysis of expression datasets GSE76703, GSE42834, and GSE144127

Analysis of previously published datasets for expression of UKHSA, Roe1/3, Sweeney3, and RISK6 biomarkers was conducted using our previously published NHP dataset GSE76703 and human datasets GSE42834 (32) and GSE144127 (72) downloaded from the National Center for Biotechnology Information (NCBI) Gene Expression Omnibus (GEO). These were imported into Agilent's GeneSpring GX version 14.9, normalized to the 75th percentile, median baseline transformed, and analyzed using various functions, e.g., hierarchical cluster analysis, analysis of variance (ANOVA), and other functions in GeneSpring GX version 14.9, using default settings. Normalized data were exported from GeneSpring GX 14.9 for further analysis using other tools, e.g., random forest analysis.

#### 2.1.3 ROC analysis and random forest modeling of TB biomarkers using expression datasets GSE76703, GSE42834, and GSE144127

Identification of top-ranking biomarkers was predicted using a combination of multivariate ROC analysis and random forest modeling using the randomForest package in R. Models were performed to classify all controls and other disease groups (i.e., cancer, pneumonia, sarcoidosis) from ATB (classifying PTB and EPTB as separate groups where possible). Data were split (two-thirds training set, one-third testing set) and modeling performed with number of predictor variables randomly sampled at each split (mtry) set to 4 and number of trees (ntree) set to 2001. Any missing values were replaced with median values for each group. Outputs included ROC curves comparing model performance, predicted class probabilities for all samples using a selected model, out-of-bag (OOB) error, predictive accuracy of biomarker models with increasing number of features, and significant features ranked by importance.

### 2.2 Prospective qPCR pilot study

#### 2.2.1 Study design, ethical approvals, sample collection, and processing

All patients recruited to this prospective, observational study at partner sites in India were recruited under approvals from both the Jawaharlal Institute of Postgraduate Medical Education and Research (JIPMER) Ethics Committee (human studies) and UKHSA, UK (India Study No. JIP/IEC/2015/11/522, UK Study No. PHE0186). A total of 45 participants were recruited to the pilot study, distributed across three groups: PTB (*n* = 15) and EPTB (*n* = 15) diagnosed with combinations of GeneXpert, MGIT, for acid-fast bacilli for smear microscopy (ZN/AFB) on sputum or tissue, chest X-ray at JIPMER Hospital, Puducherry, India, and locally recruited high-incidence region controls [(CNTRL) *n* = 15]. In accordance with the National TB Elimination Programme (NTEP) guidelines (India) at the time of recruitment, participants who were positive for ZN/AFB or using GeneXpert and exhibited radiological features consistent with PTB were classified as PTB cases. EPTB was confirmed based on positivity by one or more of the following diagnostic methods: GeneXpert, mycobacterial culture, ZN/AFB smear, or histopathological examination. Samples from healthy controls were obtained from asymptomatic individuals with no clinical history or symptoms suggestive of tuberculosis. Neither the tuberculin skin test (TST) nor the interferon-gamma release assay (IGRA) was performed; this omission has been acknowledged as a study limitation. All test results and diagnosis assessment criteria for this cohort are summarized in Table 1 and Supplementary Table S1.1. All study participants provided written informed consent. Exclusion criteria included HIV infection, current use of steroids or other immunosuppressive therapies, and pregnancy. At subject enrollment, 2.5 ml of whole venous blood was collected from each study participant in PaxGene Tubes (BD Biosciences, UK), which were stored at −80 °C prior to further processing. Samples were collected at baseline at the time of diagnosis.

**Table 1:** Clinical and diagnostic characteristics of study participants across CNTRL, EPTB, and PTB groups summarizing demographic data and diagnostic test outcomes.

**Characteristic**	**CNTRL, *n* = 15**	**EPTB, *n* = 15**	**PTB, *n* = 15**	***P*-value**
**Age** ^a, c^				0.4
	34.4 (10.48)	34.73(14.31)	40.13 (14.83)	
**Gender** ^b, d^				0.027
Female	3/15 (20%)	9/15 (60%)	3/15 (20%)	
Male	12/15 (80%)	6/15 (40%)	12/15 (80%)	
**GeneXpert** ^d^				
Negative	0/15 (0%)	1/15 (6.7%)	1/15 (6.7%)	
Positive	0/15 (0%)	9/15 (60%)	2/15 (13%)	
Not performed	15/15 (100%)	5/15 (33%)	12/15 (80%)	
**MGIT** ^d^				
Negative	0/15 (0%)	1/15 (6.7%)	2/15 (13%)	
Positive	0/15 (0%)	2/15 (13%)	0/15 (0%)	
Not performed	15/15 (100%)	12/15 (80%)	13/15 (87%)	
**Tissue histopathology** ^d^				
Clinically assessed	0/15 (0%)	9/15 (60%)	0/15 (0%)	
Not performed	15/15 (100%)	6/15 (40%)	15/15 (100%)	
**ZN/AFB sputum smear test** ^d^				
Negative	0/15 (0%)	1/15 (6.7%)	0/15 (0%)	
Positive scanty	0/15 (0%)	0/15 (0%)	1/15 (6.7%)	
Positive ungraded	0/15 (0%)	1/15 (6.7%)	0/15 (0%)	
Positive 1+	0/15 (0%)	0/15 (0%)	3/15 (30%)	
Positive 2+	0/15 (0%)	0/15 (0%)	3/15 (30%)	
Positive 3+	0/15 (0%)	1/15 (6.7%)	7/15 (47%)	
Not performed	15/15 (100%)	12/15 (80%)	1/15 (6.7%)	
**Chest X-ray** ^d^				
Available	3/15 (30%)	2/15 (13%)	15/15 (100%)	
Not taken	12/15 (80%)	13/15 (87%)	0/15 (0%)	
**LJ slope/TB culture** ^d^				
Available	0/15 (0%)	3/15 (20%)	0/15 (100%)	
Not taken	15/15 (100%)	12/15 (80%)	15/15 (100%)	

Statistically significant differences between groups were assessed using Pearson's chi-squared test, Kruskal–Wallis rank-sum test, or Fisher's exact test as appropriate. Significant *p*-values (*p* < 0.05) are given.

^a^Kruskal-Wallis rank-sum test.

^b^Pearson's chi-squared test.

^c^Mean (SD).

^d^*n*/*N* (%).

#### 2.2.2 Primers, probes, and PCR assay design

##### 2.2.2.1 qPCR design and assay development

“In-house” RT-qPCR assays were designed for 11 small-sized, TB-associated, blood/immune biomarker profile targets from different published studies including from this group: (1) *CALCOCO2, GBP1, IFIT3, IFITM3, SAMD9L*, and *SNX10* [UKHSA/India (INDUK)] (54); (2) *BATF2* [Roe1 (34)], *BATF2, GBP5*, and *SCARF1* [Roe3 (67)]; and (3) *GBP5, DUSP3*, and *KLF2* [Sweeney3 (36)]. Assays for two standard housekeeping genes, *RPL13A* and *PGK1*, were also designed for use in relative quantification, which are well-established and well-characterized, stable, endogenous control genes used in other studies (72, 73). These were confirmed as constitutively expressed across TB-infected individuals and healthy controls in our previous study (54). All so-called in-house qPCR assays were developed using Integrated DNA Technologies (IDT) PrimerQuest software. Gene sequences of each transcript variant were aligned, and assays were designed across exon–intron boundaries to prevent possible amplification of contaminating genomic DNA. Primers, hydrolysis probes, and corresponding artificial DNA gBlock fragments for each target biomarker and reference genes were purchased from IDT. Fluorescently labeled probes were selected for assays to enable them to be performed on multiple qPCR platforms. These were evaluated using the QuantStudio 5 (Applied Biosystems) and Bioline MIC (BioMolecular Systems) qPCR platforms. RNA synthesized from artificial gBlocks (IDT, UK) for each of the target genes and internal housekeeping genes were used as positive controls and run alongside qPCR assay mix (buffer only) “no template” controls (NTCs). Commercially available prequalified TaqMan gene expression primer-probe assays (Thermo Fisher Scientific) were used for the evaluation of RISK6 biomarkers (*FCGR1B, GBP2, SDR39U1, SERPING1, TRM2A*, and *TUBGCP6*) as previously described (34). Details of all primer and probe sequences, dyes, and quenchers used are given in Supplementary Table S1.2.

The 11 “in-house” designed biomarker targets were evaluated as duplex RT-qPCR assays using *RPL13A* as the housekeeping gene for relative quantification. All assays, including the prequalified Taqman RISK6 signature targets, were run as one-step RT qPCR assays using the QuantiNova Multiplex RT-qPCR kit (Qiagen), according to the manufacturer's instructions. The six single INDUK biomarker assays were then translated into two quadruplex assays (3 × targets and including control genes) for further evaluation of minimal-size assay formats, by substitution of hydrolysis probe fluorophore labels for some of the targets from FAM to Texas Red or Cy5 to allow for multiplexing (see Supplementary Table S1.3). *RPL13A* was included as a housekeeping gene in quadruplex reaction with *GBP1, CALCOCO2*, and *SNX10, PGK1* was included in the quadruplex reaction with *IFIT3, IFITM3*, and *SAMD9L*, fluorescently labeled with Hex. Both housekeeping genes were used in separate assay wells (i.e., using two assay configurations per sample) due to constraints in multiplexing capability ( ≤ 4), from limitations imposed by one of the qPCR platforms used for evaluation (i.e., the four-laser channel Bioline MIC). This was done to standardize biomarker target expression in each sample, to enable accurate comparison of target expression values between samples, effectively to reduce technical variations in assay numeric data outputs due to RNA loading and therefore enhance accuracy and robustness. All primer/probe design, assay development, and analyses were conducted following guidance for minimum information for the publication of quantitative real-time PCR experiment (MIQE) guidelines (68).

##### 2.2.2.2 Experimental evaluation of qPCR assays using artificial RNA constructs

Assays were evaluated and optimized to determine limits of detection (LOD), stability, and robustness using standard curves ranging from 10^7^ to 10^0^ copies of RNA synthesized from the respective artificial gBlock fragments using the SuperScript III First-Strand Synthesis System (Thermo Fisher Scientific). Analytical performance parameters, including sensitivity, efficiency, linearity, and precision, were assessed to determine the suitability of assays using the Quantstudio 5 and Bioline MIC platforms. Each assay was required to show excellent correlation (*R*^2^ > 0.99) and an efficiency nearing 100% (90–110%), to be considered fit for purpose before further development and clinical evaluation. Quality control and data analysis were initially conducted using QuantStudio Design & Analysis Software version 1.5.1 or MIC qPCR software, and data were then exported and analyzed further using ExpressionSuite (Thermo Fisher Scientific).

#### 2.2.3 Purification of RNA and RT qPCR validation of differentially expressed biomarkers in a new cohort clinical prospective study

Total RNA was purified from blood samples using the PaxGene Blood RNA kit (Qiagen), according to the manufacturer's instructions. The purity of mRNAs was assessed using a Nanodrop ND-1000 spectrophotometer (Thermo Fisher Scientific, EUA) prior to use. Duplex and quadruplex RT qPCR analyses were performed for all samples over multiple runs on a QuantStudio 5 (Applied Biosystems) platform using the QuantiNova Multiplex RT-PCR kit (Qiagen). Laboratory personnel were blinded to participant diagnostic status during assay processing and analysis. RNA was diluted 1:5 in DEPC-treated water and added to individual wells of a PCR microtiter plate in 5-μl volumes in a final reaction volume of 20 μl. Each assay reaction was run in triplicate, and RT-qPCRs were performed under the following cycling conditions: reverse transcription: 10 min at 50 °C; heat activation: 2 min at 95 °C; denaturation: 5 s at 95 °C; annealing/extension: 30 s at 60 °C; denaturation and annealing/extension steps repeated for 40 cycles.

#### 2.2.4 Relative quantification

For all individual INDUK, Roe3, and Sweeney3 Signature biomarker target duplex assays, relative quantification (RQ) was calculated using a comparative Ct (ΔΔCt) method, as a measure of relative fold change compared to the housekeeping gene *RPL13A*, for normalization purposes. Samples were grouped according to sample type, i.e., CNTRL, EPTB, PTB (and also combined as ATB), and fold change calculated as 2(-ΔΔCT), as relative expression of biomarkers in the TB disease groups compared to CNTRLs. Due to variability in reaction efficiencies between target and reference genes, the theoretical doubling of amplicon was used, and a reaction efficiency of 100% was assumed. RQ values were log2-transformed before further statistical analysis. CNTRLs were used as calibrators for ΔΔCT calculations. For calculation of relative quantification in quadruplex (4PLEX) assays (Tube1; *CALCOCO2, GBP1, SAMD9L* plus *RPL13A*, Tube2; *IFIT3, IFITM3, SNX10* plus *PGK1*), expression levels of target genes were compared to reference genes *RPL13A* and *PGK*1 using the *Vandesompele* method (74), utilizing the geometric means of the housekeeping genes to normalize expression values. After data export, RQ values were calculated for all INDUK and Roe1, Roe3 and Sweeney3 biomarker targets individually as a measure of fold change on biologically grouped data. Relative gene expression values were log2-transformed prior to further analysis. RQ was not used for the calculation of RISK6 biomarkers; instead, raw data values (Cq) were analyzed using the previously published RISK6 score algorithm as described previously (28). The following algorithms were used to evaluate the performance of the Roe3, Sweeney3, and RISK6 signatures, taken from previous publications:

Roe3: (*SCARF1*+*GBP5*+*BATF2*)/3 (66)

Sweeney3: ([*GBP5*+*DUSP3*]/2)-*KLF2* (36)

RISK6: GEOMEAN(*SDR39U1, TRM2A, TUBGCP6*)-GEOMEAN(*FCGR1B, GBP2, SERPING1*) (28, 50).

#### 2.2.5 Receiver operating characteristic/area under the curve and performance analysis

Receiver operating characteristic (ROC) curve analyses were performed on log2-transformed RQ data to quantify performance on normalized Cq values for the RISK6 signature. ROC curves were plotted using R × 64 3.4.0 software using the ROCR package (113), the ROC analysis tool of Sigmaplot 12.0 or Graphpad Prism 8.0. The accuracy of each candidate biomarker and signature was measured by calculating AUROC values. Cut-off values were predicted by measuring the optimal accuracy of the curve, from which the sensitivity and specificity of each biomarker/biomarker panel test were determined. Cut-offs were selected, where possible, to obtain appropriate sensitivity and specificity according to the TPP requirements listed below. If TPPs could not be met, cut-offs were selected that showed the optimal sensitivity/specificity.

1. TPP 2014 Confirmatory Test
  - Optimal: 98% sensitivity, 98% specificity (PTB);
  - 80% sensitivity, 98% specificity (EPTB)
  - Minimal: 65% sensitivity, 98% specificity
2. TPP 2014 Triage Test
  - Optimal: 95% sensitivity, 80% specificity
  - Minimal: 90% sensitivity, 70% specificity
3. TPP 2024 Confirmatory Test Update
  - Optimal: 95% sensitivity, 98% specificity
  - Low complexity: 85% sensitivity, 98% specificity
  - Near POC: 75% sensitivity, 98% specificity
  - POC: 65% sensitivity, 98% specificity

Combined biomarker signatures were also assessed to determine whether they showed improved discrimination compared with single biomarkers, between control and TB-infected groups. Simple algorithms consisting of biomarkers combined additively or averaged to generate a single composite qPCR-derived signature score were assessed by ROC analysis, and the diagnostic performance was further assessed using sensitivity, specificity, cut-off values, and likelihood ratios. Select biomarker panel configurations were also compared against WHO TPP requirements for triage and confirmatory test minimum and optimum requirements. Outputs were depicted graphically using either Sigmaplot 12.0, GraphPad PRISM 9.0, or Biorender (https://www.biorender.com/).

#### 2.2.6 Other statistical analyses for determination of clinical performance

All generated RQ data were log2-transformed, and non-parametric Mann–Whitney *U* tests were performed between groups using multiple testing correction (75). Diagnostic accuracy was assessed and measured using sensitivity, specificity, positive predictive value (PPV), negative predictive value (NPV), positive likelihood ratio (LR+), and negative likelihood ratio (LR–) using GraphPad PRISM 9.0.

#### 2.2.7 Decision curve analysis

To assess the clinical utility of candidate biomarker signatures for TB triage, we performed decision curve analysis (DCA) using the rmda package in R (version 4.4.2) (76). Models evaluated included individual INDUK biomarkers and biomarker signatures Roe1, INDUK2, and INDUK3 and a unified combination of INDUK3+Roe1 (INDUK3.Roe1). Each model was derived from previously validated multiplex RT-qPCR data and applied to a test dataset with binary TB outcome labels [ATB vs. Non-TB (CNTRL)]. DCA was conducted by estimating net benefit across a range of threshold probabilities from 0.01 to 1.0, representing the probability at which a clinician would choose to refer a patient for confirmatory TB testing. Net benefit was calculated using the standard formula


$$\begin{array}{l} Net\;Benefit=\frac{True\;Positives}{n}-\frac{False\;Positives\;}{n}\times \;\frac{p_{t}}{1-p_{t}} \end{array}$$

where *n* is the total sample size and *p*_*t*_ is the threshold probability. Standard “treat all” and “treat none” strategies were included as reference comparators. This analysis enables estimation of clinical consequences of implementing each model in settings with constrained diagnostic capacity. Results were visualized with net benefit plotted against threshold probability and corresponding cost: benefit ratios to reflect real-world trade-offs in triage decision-making.

#### 2.2.8 Scenario analysis

Scenario analysis was conducted to estimate PPV and NPV of the INDUK3 assays across varying hypothetical prevalence levels (5%, 10%, 20%, and 41%) (77). The objective was to evaluate how varying disease prevalence impacts the predictive accuracy of the test while assuming constant sensitivity and specificity. A baseline PPV of 91.89% and NPV of 62.5% at a 31% prevalence was used to back-calculate test characteristics of sensitivity and specificity. Using standard diagnostic equations and numerical approximation, test sensitivity and specificity were estimated to be approximately 70% and 95%, respectively. These values were then applied to estimate PPV and NPV at four additional prevalence levels: 5%, 10%, 20%, and 41% using What-If (Data Table) Analysis in Microsoft Excel. Calculations were based on the following formulas (78):


$$\begin{array}{l} \text{PP}\text{V}=\frac{Se\times P}{Se\times P+\left(1-Sp\right)\times \left(1-P\right)} \end{array}$$

$$\begin{array}{l} \text{NP}\text{V}=\frac{Sp\times \left(1-P\right)}{Sp\times \left(1-P\right)+\left(1-Se\right)\times P} \end{array}$$

where:

*Se* = sensitivity

*Sp* = specificity

*P* = disease prevalence

## 3 Results

### 3.1 STRING functional enrichment and GeneSpring analysis of TB biomarkers

#### 3.1.1 STRING functional enrichment analysis

**Figure 1:**
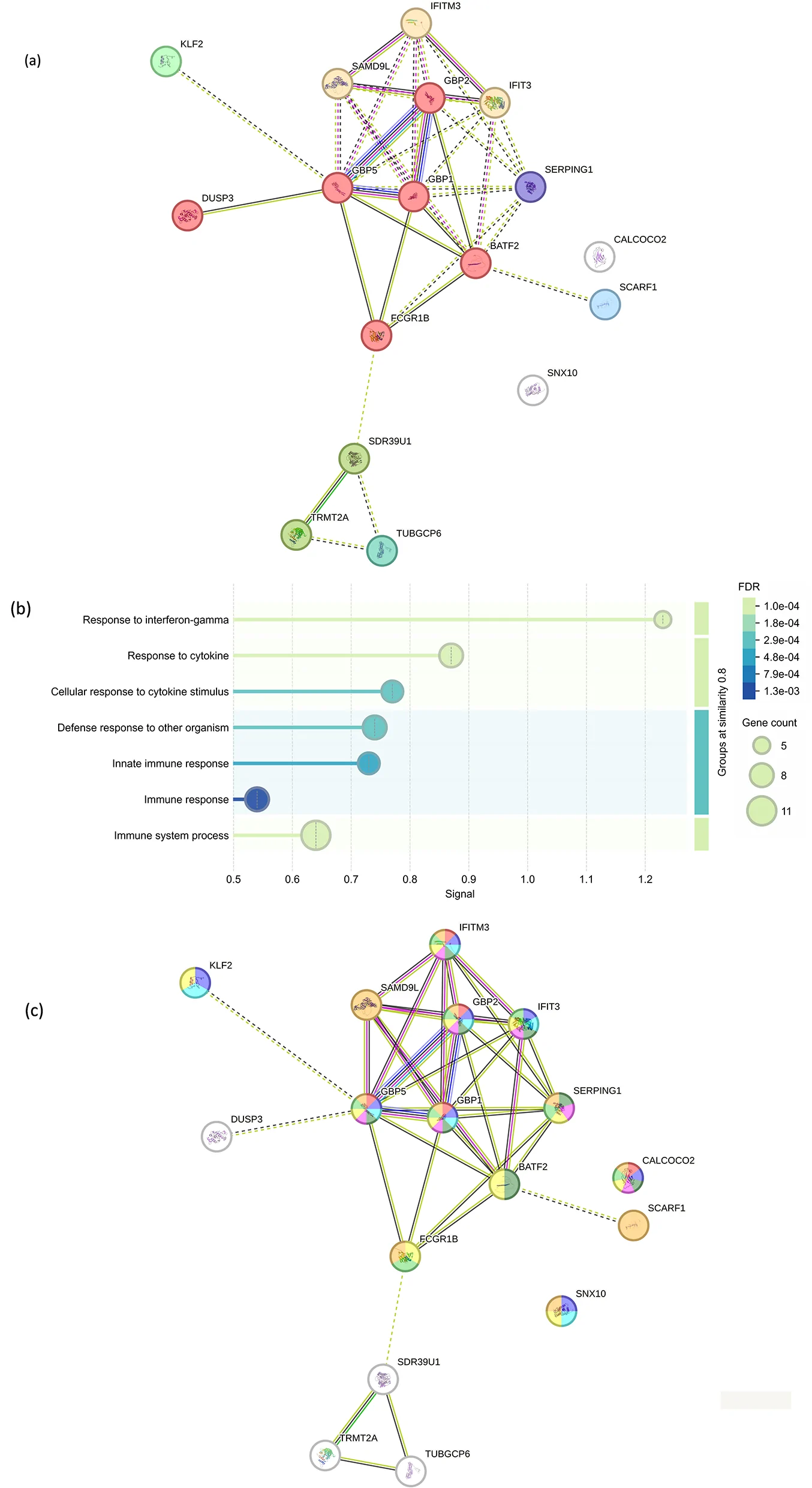
**(a)** STRING K-means clustering map of all UKHSA, Roe1/3, Sweeney3, and RISK6 biomarker targets; Cluster 1 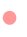 (guanylate binding protein cluster), Cluster 2 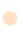 (mixed; IFN α/β signaling, guanylate binding protein cluster), Cluster 3 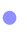 (*SERPING1* single cluster), Cluster 4 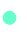 (*KLF2* single cluster), Cluster 5 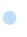 (*SCARF1* single cluster), Cluster 6 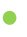 (*SDR39U1* and *TRMT2A* cluster), Cluster 7 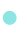 (*TUBGCP6* single cluster), no cluster association 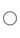 (*CALCOCO2* and *SNX10* single entity clusters). **(b)** Breakdown of biomarker functional associations by gene count and significance. **(c)** Breakdown of biomarker functional associations by pathway and GO term; IFNγ (GO:0034341) 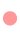, response to cytokine (GO:0034097)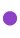, response to cytokine stimulus (GO:0071345) 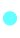, defense response to other organism (GO:0098542) 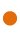, innate immune response (GO:0045087) 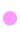, immune system process (GO:0002376) 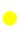, immune response (GO:0006955) 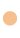, cytoplasmic vesicle (GO:0031410) 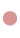, no pathway association 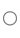.

All study biomarker entities were combined and analyzed for STRING-derived PPI using STRING version 12.0 as described. The PPI network of *H. sapiens* immune-related genes contained 17 nodes and 37 edges (expected number of no association edges threshold = 1, indicating the biomarker network had significantly more interactions than expected by chance) at a confidence threshold of ≥0.7, reflecting a moderately interconnected host immune network. The average local clustering coefficient was 0.724, average node degree 4.35, and PPI enrichment *p*-value < 1.0^−16^. Several regions of dense connectivity were observed, indicating potential functional modules (all PPI interaction data are given in Supplementary Table S1.4 and associated processes in Supplementary Table S1.5). Visualization confirmed modular organization with dense intracluster connectivity and limited intercluster overlap (Figure 1). Several hub proteins, including *GBP1, GBP2, GBP5, BATF2*, and *IFIT3*, showed a high degree of betweenness centrality, suggesting potential key roles in mediating immune responses to *M. tuberculosis*. These results demonstrate the modular structure of the host immune protein network and identified these candidate hub genes that may contribute to immune regulation during tuberculosis infection.

K-means clustering (group setting *n* = 7) partitioned the network into four distinct clusters based on protein interaction profiles (Figure 1a and Supplementary Table S1.6). Cluster 1 (guanylate-binding protein, C-terminal) included *BATF2, DUSP3, GBP1, GBP2, GBP5*, and *FCGR1B* exhibited strong co-expression with a PPI enrichment *p*-value < 1.0^−16^, suggesting coordinated regulation in antiviral and interferon-mediated responses. Cluster 2 (mixed, including interferon alpha/beta signaling, and guanylate-binding protein, C-terminal) contained *IFIT3, IFITM3*, and *SAMD9L*, with a PPI enrichment *p*-value of 1.72^−6^, involved in interferon-induced antiviral responses, highlighting their functional connectivity in host defense pathways. Cluster 3 included *SDR39U1* and TRMT2A with a PPI enrichment *p*-value of 0.00144, which also more loosely associated with *TUBGCP6* (Cluster 5), reflecting a less-characterized or indirect immune interactions. *KLF2* (Cluster 4) associates with Cluster 1 through *GBP5* (PPI enrichment score *p*-value of 0.077), *SCARF1* associates with Cluster 1 through *BATF2* (PPI enrichment score *p*-value of 0.098), and *SERPING1* associates with Cluster 1 through interactions with *BATF2, GBP1, GBP2, GBP5*, and *FCGR1B* (average PPI enrichment score *p*-value of 0.096). *SCARF1* associates with Cluster 2 through *IFIT3* (PPI enrichment score *p*-value of 0.055) and *IFITM3* (average PPI enrichment score *p*-value of 0.068). *CALCOCO2* and *SNX10* did not form an associative relationship with any of the other 15 biomarkers.

The majority of the biomarkers identified in this study were significantly associated with Gene Ontology (GO) terms related to immune processes (Figures 1b, c), including interferon-gamma (IFNγ) response (GO:0034341), response to cytokine (GO:0034097), response to cytokine stimulus (GO:0071345), defense response to other organism (GO:0098542), innate immune response (GO:0045087), immune system process (GO:0002376), immune response (GO:0006955), and cytoplasmic vesicle (GO:0031410). A subset of genes, *DUSP3, SDR39U1, TRMT2A*, and *TUBGCP6*, did not map to any known GO biological process. Most biomarkers were associated with multiple GO terms (≥2) associated with diverse combinations of overlapping immune and cellular functions. Notable exceptions included *SAMD9L* and *SCARF1*, each mapping to a single process (cytoplasmic vesicle), and *BATF2*, which mapped to two processes (immune response and immune system process). *KLF2* and *FCGR1B* were each mapped to three GO categories: immune system process, response to cytokine stimulus/cytokine, and immune response/cytoplasmic vesicle, respectively. Cluster 1 gene entities *GBP1, GBP2*, and *GBP5, IFITM3* in Cluster 2, and *CALCOCO2* were identified as IFNγ-regulated (GO:0034341). The remaining Cluster 1 and 2 entities appear to not be IFNγ-regulated, including *IFIT3*, which may be influenced by other factors, e.g., type I interferons (IFNα/β). Overall, these results suggest that although the biomarkers examined exhibit shared enrichment in immune-related pathways and overlapping GO annotations, they display functional diversity that may reflect specialization within immune regulatory networks.

#### 3.1.2 Analysis of TB biomarkers using NHP expression dataset GSE76703

**Figure 2:**
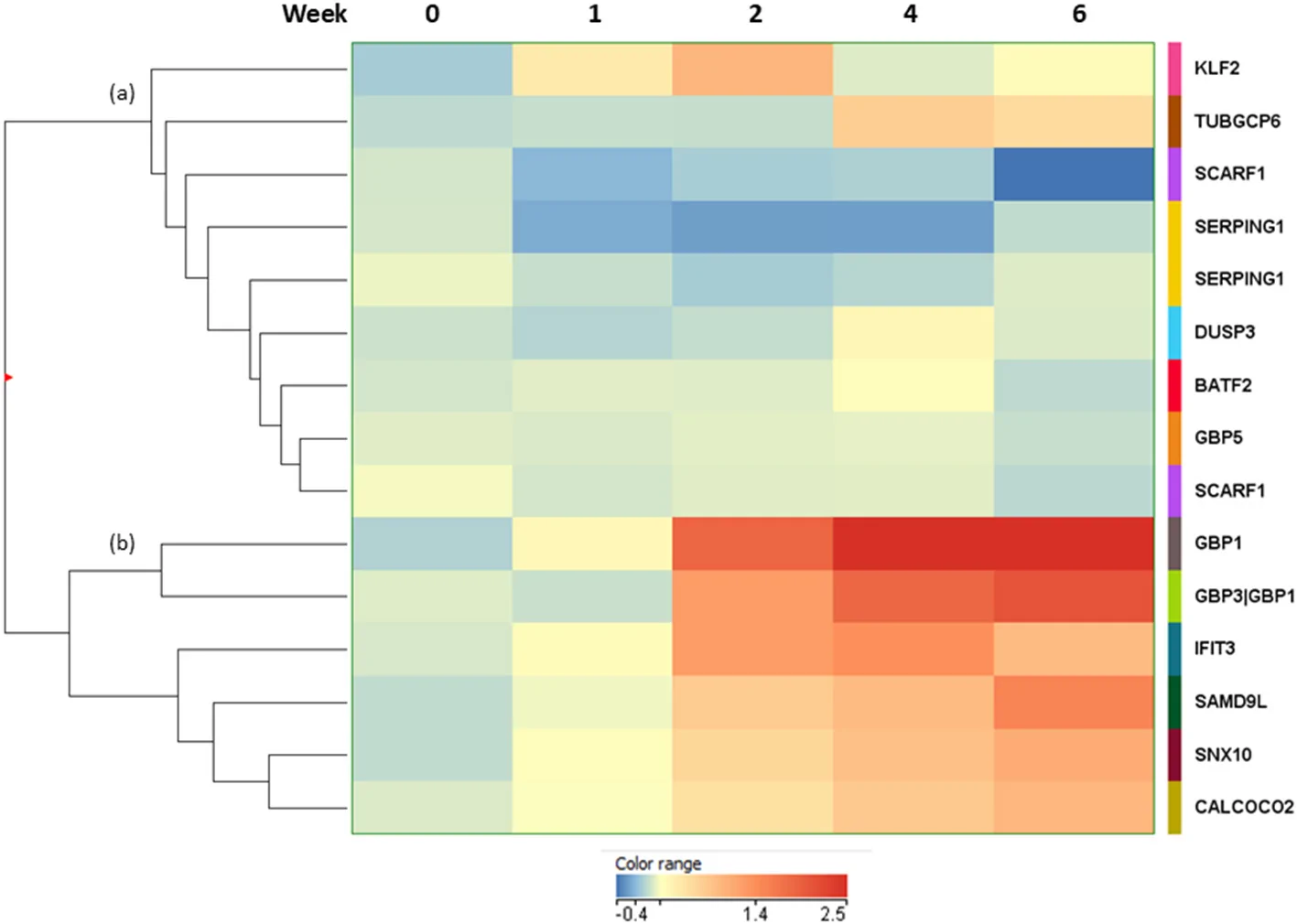
Heatmap of UKHSA, Roe1/3, Sweeney3, and RISK6 biomarker gene targets from dataset GSE76703 across timepoints from Week 0 (prebleed) to Week 6: **(a)** cluster displaying variable expression across timepoints; **(b)** cluster displaying incremental, sustained expression across timepoints.

GeneSpring analysis of the NHP transcriptomic dataset GSE76703 was conducted using a predefined gene list comprising only the relevant biomarkers from the UKHSA, Roe1/3, Sweeney3, and RISK6 signatures. Biomarkers not represented on the array (*IFITM3, FCGR1B, GBP2, SDR39U1*, and *TRMT2A*) were excluded from the analysis. Results are provided in Supplementary Table S1.4, and visualized as a heatmap in Figure 2.

ANOVA (*p* < 0.05, corrected) identified the following biomarkers as significantly differentially expressed across time points: *GBP1, GBP3|GBP1, SNX10, CALCOCO2, SAMD9L, KLF2, TUBGCP6, IFIT3, SCARF1, SERPING1.1, DUSP3*, and *SERPING1.2, BATF2, SCARF1*, and *GBP5* were not significantly different. Temporal variation in expression was observed, with biomarkers clustering into two main expression profiles: (a) transient or weak early expression and (b) sustained and incremental expression from Week 1 onward. Notably, *KLF2* showed a dynamic pattern, upregulated until Week 2, followed by downregulation at Week 4, and partial recovery at Week 6. *BATF2* and *DUSP3* exhibited delayed, weak expression peaking around Week 4, inversely related to *KLF2*. *TUBGCP6* showed modest upregulation starting at Week 4, while *IFIT3* demonstrated sustained expression with a slight decrease by Week 6. These patterns encompassed biomarkers from diverse immune-related functional categories (e.g., *IFN-*γ signaling, cytokine response), as shown in Figure 1. No specific cell-type origin could be confidently assigned. These findings indicate that the diagnostic performance of immune biomarkers may vary depending on disease stage or phase (e.g., early, established, or resolving TB). To further prioritize biomarkers, random forest modeling was performed across prebleed, Week 1, Week 2, Week 4, and Week 6 samples, achieving an OOB error rate of 0.178 (Supplementary Figure S2.1A). Top-ranking features included *GBP1, KLF2*, and *SNX10*, as shown in Supplementary Figure S2.1B.

#### 3.1.3 Analysis of TB biomarkers using human expression datasets GSE42834 and GSE144127

**Figure 3:**
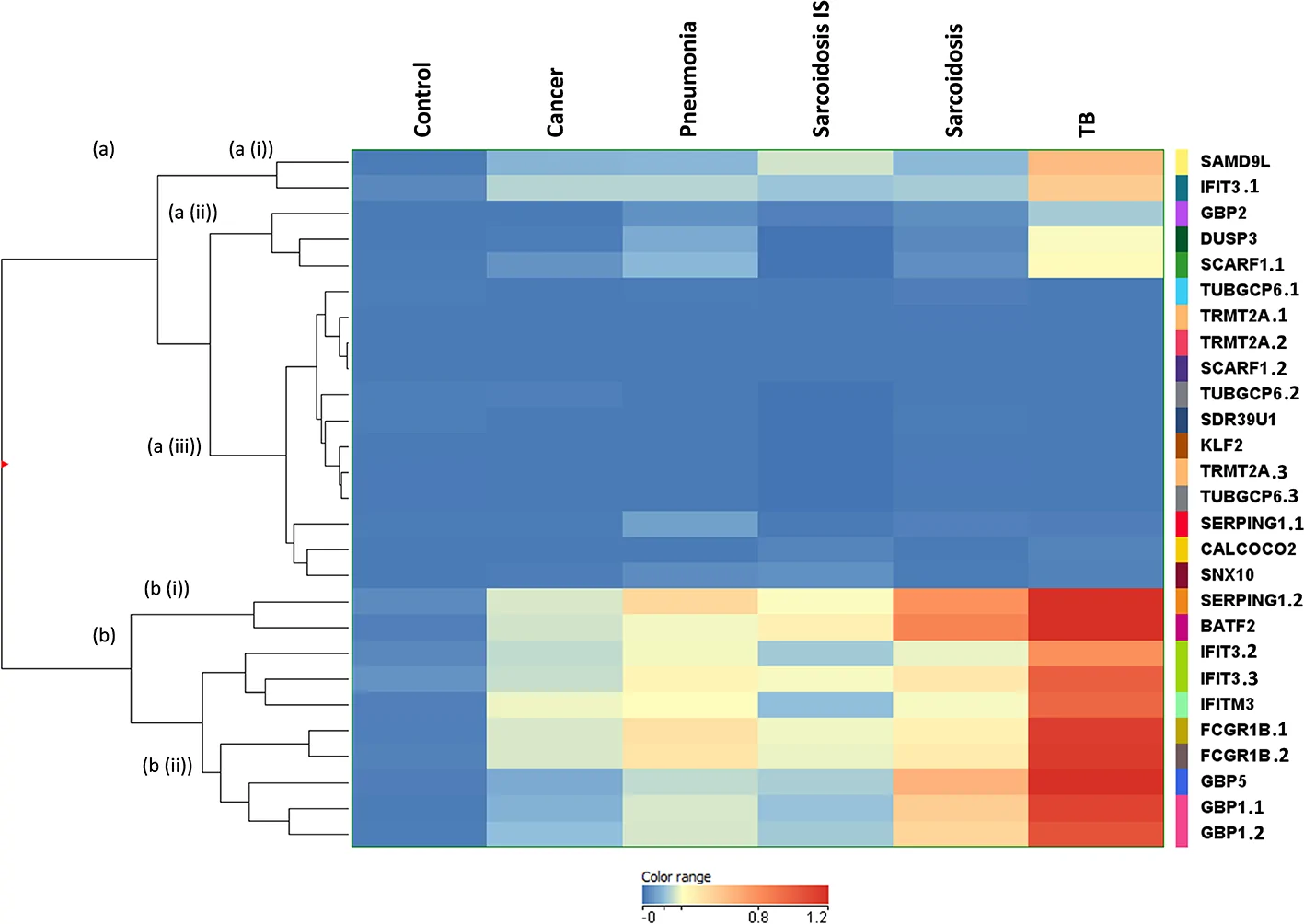
Heatmap of UKHSA, Roe1/3, Sweeney3, and RISK6 biomarker gene targets from dataset GSE42834 across different diseases **[a(i)]** cluster displaying some expression across diseases but highest in TB, **[a(ii)]** cluster displaying some expression across pneumonia and sarcoidosis but highest in TB, **[a(ii)]** cluster displaying low expression across all diseases, **[b(i)]** cluster displaying some expression across diseases but highest in both sarcoidosis and TB, **[b(ii)]** cluster displaying some expression across diseases but highest in TB.

**Figure 4:**
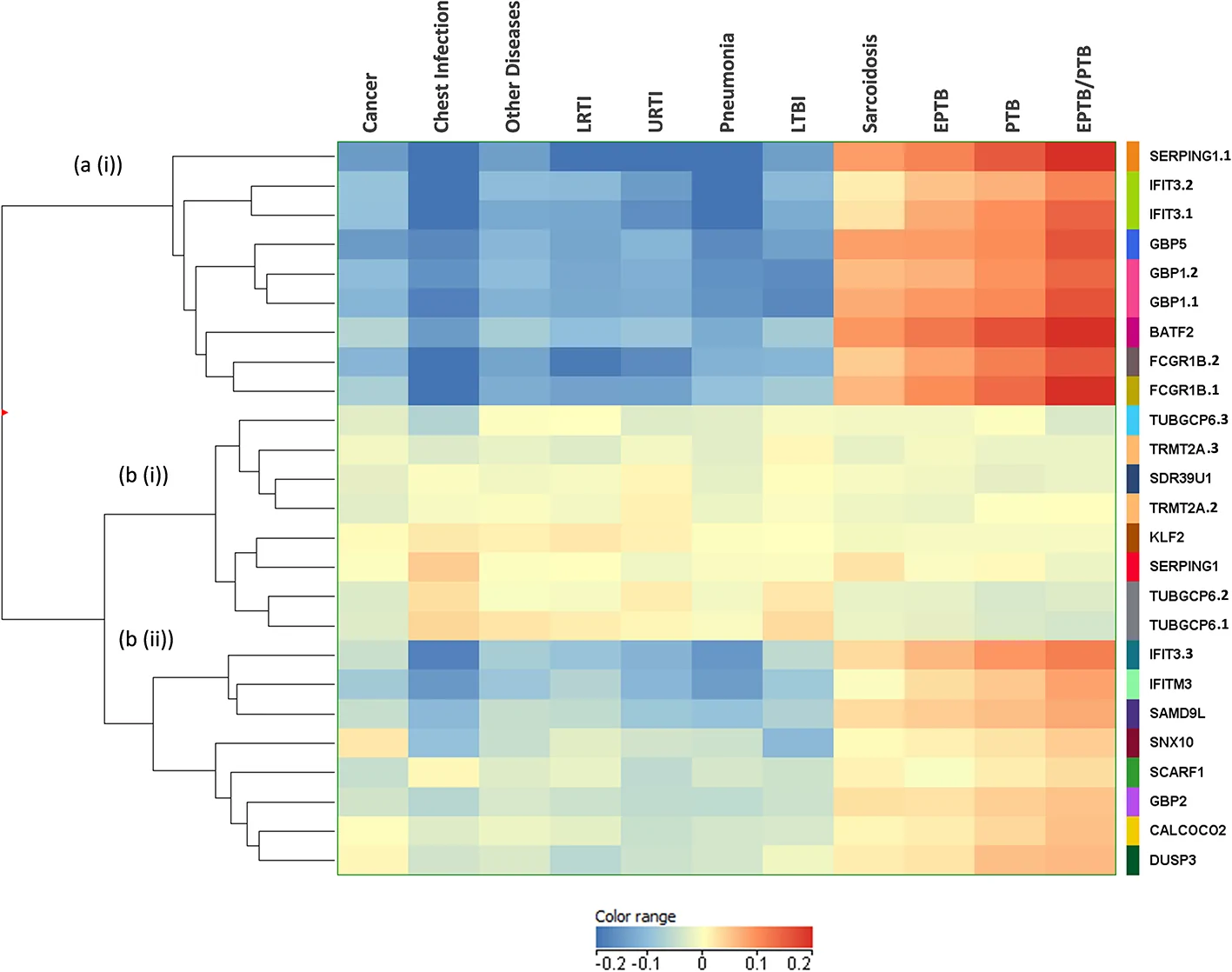
Heatmap of UKHSA, Roe1/3, Sweeney3, and RISK6 biomarker gene targets from dataset GSE144127 across different diseases: **[a(i)]** cluster displaying generalized low expression across other diseases but high expression in sarcoidosis, EPTB, PTB, and EPTB/PTB; **[b(i)]** cluster displaying some variable expression across other diseases but relatively lower expression in EPTB, PTB, and EPTB/PTB and sarcoidosis except for *SERPING1*; **[b(i)]** cluster displaying some variable expression across other diseases but relatively higher expression in EPTB, PTB, and EPTB/PTB and sarcoidosis.

GeneSpring analysis of the datasets GSE42834 and GSE144127 were analyzed using a predefined gene list consisting only of the study's selected biomarkers. Full results are provided in Supplementary Tables S1.5, S1.6, with heatmap visualizations in Figures 3, 4, respectively. These datasets were selected because they include samples from individuals with TB as well as other infectious or inflammatory diseases, enabling a broader assessment of biomarker specificity.

ANOVA analysis (*p* < 0.05, corrected) identified numerous significantly differentially regulated biomarkers ordered by significance when comparing cancer vs. all other disease groups (GSE42834) and controls vs. all other disease groups (GSE144127), as presented in Table 2. Significant overlap was observed between the two datasets, suggesting consistency in biomarker expression patterns across independent cohorts. In GSE42834 (Figure 3), biomarker expression clustered into two main groups (a and b), with subclusters identified within group (a). Subclusters a(i) and a(ii) contained biomarkers such as *SAMD9L, IFIT3, GBP2, DUSP3*, and *SCARF1*, showing preferential expression in TB relative to other diseases. Subcluster a(iii) included biomarkers with minimal expression across all groups, with the exception of *SERPING1*, which showed elevated expression in pneumonia. Cluster b included biomarkers with broader expression across diseases such as cancer, pneumonia, and sarcoidosis. *FCGR1B* and *SERPING1* were highly expressed in pneumonia, sarcoidosis, and TB, while *BATF2* showed stronger expression in sarcoidosis and TB. *IFIT3* and *IFITM3* were more specific to TB, while *GBP1* and *GBP5* were elevated in both sarcoidosis and TB, but higher in TB.

**Table 2:** Significantly differentially expressed genes in control/cancer vs. all other disease groups in Public Datasets GSE42834 and GSE144127.

**Rank**	**GSE42834**		**GSE144127**	
	**Probe ID**	**Gene symbol**	**Probe ID**	**Gene symbol**
1	ILMN 1690241	*BATF2*	ILMN 1670305	**SERPING1*.1*
2	ILMN 2114568	*GBP5*	ILMN 2114568	*GBP5*
3	ILMN 1670305	**SERPING1*.2*	ILMN 1690241	*BATF2*
4	ILMN 1701114	**GBP1*.1*	ILMN 2261600	**FCGR1B*.2*
5	ILMN 2261600	**FCGR1B*.2*	ILMN 1701114	*GBP1*
6	ILMN 2391051	**FCGR1B*.1*	ILMN 2391051	**FCGR1B*.1*
7	ILMN 2148785	**GBP1*.2*	ILMN 2148785	**GBP1*.2*
8	ILMN 1805750	*IFITM3*	ILMN 1774077	*GBP2*
9	ILMN 1664543	*IFIT3.3*	ILMN 1701789	*IFIT3.2*
10	ILMN 1799467	*SAMD9L*	ILMN 1664543	*IFIT3.3*
11	ILMN 1701789	*IFIT3.2*	ILMN 1799467	*SAMD9L*
12	ILMN 1797522	*DUSP3*	ILMN 1805750	*IFITM3*
13	ILMN 2390946	**SCARF1*.1*	ILMN 2239754	*IFIT3.1*
14	ILMN 1774077	*GBP2*	ILMN 1797522	*DUSP3*
15	ILMN 2239754	*IFIT3.1*	ILMN 1755504	*CALCOCO2*
16	ILMN 1735930	*KLF2*	ILMN 2170949	*SNX10*
17	ILMN 1711272	**SERPING1*.1*	ILMN 2390946	*SCARF1*
18	ILMN 2170949	*SNX10*	ILMN 1766803	**TUBGCP6*.1*
19	ILMN 1671913	*TRMT2A.1*	ILMN 1735930	*KLF2*
20	ILMN 1766803	**TUBGCP6*.1*	ILMN 2412571	**TUBGCP6*.2*
21	ILMN 1755504	*CALCOCO2*		

Genes listed met the threshold for statistical significance (*p* < 0.05) across comparisons.

In GSE144127 (Figure 4), two primary clusters (a and b) were observed. Cluster a and subcluster b(ii) included biomarkers with strong expression in PTB, EPTB, EPTB/PTB, and sarcoidosis. Subcluster b(i) included biomarkers that were largely downregulated in these groups, with *SERPING1* being a notable exception, showing elevated expression in sarcoidosis. Together, these analyses confirm the differential expression of several biomarkers across TB and non-TB disease states and highlighted a subset with potential for discriminating TB from other inflammatory conditions.

#### 3.1.4 Random forest modeling of human datasets GSE42834 and GSE144127

To further evaluate classification performance across disease states, random forest classification models were applied to the two independent human gene expression datasets, GSE42834 and GSE144127, comparing PTB and EPTB against other disease groups, including sarcoidosis, pneumonia, cancer, and chest infections.

Across both datasets, classification of non-TB diseases achieved AUROC scores exceeding 0.8. However, discrimination between TB (both PTB and EPTB) and sarcoidosis remained challenging, likely due to overlapping transcriptional profiles. The most informative biomarkers for this comparison included *SERPING1, FCGR1B, GBP2, SAMD9L*, and *IFIT3*. In GSE42834, the random forest model demonstrated robust classification performance, achieving high AUROC values and a moderate OOB error rate of 0.321. Feature importance analysis identified *BATF2, SERPING1, FCGR1B, GBP5, IFITM3, IFIT3*, and *GBP1* as the top-ranked genes for distinguishing TB from controls and other diseases. Notably, *FCGR1B* and *IFITM3* were most informative in differentiating TB from sarcoidosis, aligning with ANOVA-based rankings (Supplementary Figure S2.3). In GSE144127, the model yielded an OOB error rate of 0.371, indicating moderate predictive accuracy (Supplementary Figure S2.4A). The most discriminative features included *GBP5, GBP1, BATF2, SERPING1, FCGR1B*, and *GBP2* and secondary array probes for *GBP1, FCGR1B*, and *IFIT3*. These rankings largely agreed with ANOVA results, with minor differences in rank order (Supplementary Figure S2.4B).

Importantly, while the most discriminative features varied between datasets in rank order, there are common features e.g., *BATF2* and *GBP5* demonstrated consistently strong discriminatory performance, in distinguishing TB from other phenotypically similar conditions. Evaluation of published TB transcriptional signature biomarkers from the INDUK, Roe1/3, Sweeney3, and RISK6 sets revealed variable importance depending on the disease context and cohort composition. These findings underscore the potential value of integrating biomarkers from heterogeneous sources, supporting the concept of developing composite gene expression signatures to enhance diagnostic precision.

### 3.2 Demographic and diagnostic characteristics of pilot study participants

A total of 45 participants were included in this pilot study, comprising 15 individuals in each of three groups: CNTRL, EPTB, and PTB. Table 1 summarizes demographic data (gender, age) and diagnostic test outcomes (GeneXpert, MGIT, ZN/AFB, radiology, and chest X-ray availability) for each group. Data are presented as count/total (percentage) for categorical variables and mean (standard deviation) for continuous variables. Diagnostic categories include results from GeneXpert MTB/RIF assay, MGIT culture, ZN/AFB smear microscopy, and radiological assessments (given in full in Supplementary Table S1.1). The mean age across groups was comparable (control: 34.4 ± 10.5 years; EPTB: 34.7 ± 14.3 years; PTB: 40.1 ± 14.8 years; *p* = 0.40). However, significant differences in gender distribution were observed (*p* = 0.027), with a higher proportion of females in the EPTB group [60% (9 out of 15)] compared to the PTB and control groups [20% each (3 out of 15)].

All patients were diagnosed according to the National TB Elimination Programme (NTEP) guidelines (India) at the time of recruitment. For diagnosis of TB in PTB patients, ZN/AFB smear positivity and chest X-ray were the principal diagnostic methods of choice. Ninety-three percent (14 out of 15) of patients had ZN/AFB-positive results, the remaining patient was positive using GeneXpert [6.7% (1 out of 15)]. ZN/AFB results demonstrated varying grades of positivity (scanty/ungraded through to 1+, 2+ % 3+). All PTB patients (15 out of 15) had chest X-Ray radiology performed, all of which exhibited clinical profiles indicative of TB pathology. Culture was not performed for all PTB samples. For the EPTB patients, GeneXpert was the diagnostic methodology of choice (67.7%) and/or variously MGIT (19.7%), ZN/AFB (19.7%), or culture (19.7%). Histopathology on tissue samples was also performed, with 9 out of 15 patients having results consistent with TB pathology (90% of EPTB patients tested), MGIT was performed on 20% (3 out of 15) EPTB patients and 13.3% (2 out of 15) PTB patients. No diagnostic testing was performed on healthy controls; however, chest X-Ray radiology was available for 20% (3 out of 15) participants with no indication of TB pathology.

### 3.3 Discriminatory performance of individual high-performing biomarkers using duplex qPCR

#### 3.3.1 Evaluation of biomarker qPCR assay performance and ROC curve analysis

Using the newly designed duplex qPCR assays, significant differential expression was observed in 8 out of 11 candidate biomarkers in comparisons of TB disease groups to CNTRL. Specifically, *BATF2, GBP1, GBP5, IFIT3, IFITM3, SAMD9L, SCARF1*, and *SNX10* demonstrated robust diagnostic potential, each exceeding an AUROC of 0.8 and exhibiting fold-change values greater than 2 in at least one disease-control comparison (Table 3). Among these, *BATF2* showed the highest diagnostic accuracy for distinguishing TB from CNTRLs (PTB vs. CNTRL: AUROC = 1.0, *p* < 1 × 10^−4^, FC = 4.31, EPTB vs. CNTRL: AUROC = 0.796, *p* = 5.8 × 10^−3^, FC = 2.06, ATB vs. CNTRL: AUROC = 0.898, *p* < 1 × 10^−4^, FC = 3.19). *IFIT3* emerged as the most effective biomarker for identifying EPTB and ATB across all disease groups (PTB vs. CNTRL: AUROC = 0.960, *p* < 1 × 10^−4^, FC = 2.83, EPTB vs. CNTRL: AUROC = 0.911, *p* < 1 × 10^−4^, FC = 2.38, ATB vs. CNTRL: AUROC = 0.936, *p* < 1 × 10^−4^, FC = 2.61). Comprehensive analyses of individual biomarker performance metrics are provided in Supplementary Table S3.2.

**Table 3:** Diagnostic performance of individual biomarkers for distinguishing PTB, EPTB, and ATB from CNTRL, as assessed by ROC analysis and fold-change evaluation.

**Biomarker**	**PTB**			**EPTB**			**ATB**		
	**AUROC**	***P*** **-value**	**Log FC**	**AUROC**	***P*** **-value**	**Log FC**	**AUROC**	***P*** **-value**	**Log FC**
*BATF2*	1.0	< 1 × 10^−4^	4.31	0.796	5.8 × 10^−3^	2.06	0.898	< 1 × 10^−4^	3.19
*GBP1*	0.969	< 1 × 10^−4^	3.14	0.884	3.0 × 10^−3^	1.97	0.927	< 1 × 10^−4^	2.56
*GBP5*	0.929	< 1 × 10^−4^	2.49	0.871	5.0 × 10^−3^	1.71	0.900	< 1 × 10^−4^	2.10
*IFIT3*	0.960	< 1 × 10^−4^	2.83	0.911	< 1 × 10^−4^	2.38	0.936	< 1 × 10^−4^	2.61
*IFITM3*	0.924	< 1 × 10^−4^	2.03	0.747	2.13 × 10^−3^	1.22	0.836	3.0 × 10^−3^	1.63
*SAMD9L*	0.978	< 1 × 10^−4^	2.18	0.889	3.0 × 10^−3^	2.34	0.933	< 1 × 10^−4^	2.26
*SCARF1*	0.822	2.6 × 10^−3^	2.02	0.809	3.9 × 10^−3^	2.29	0.816	6.0 × 10^−4^	2.16
*SNX10*	0.818	3.0 × 10^−3^	1.20	0.796	5.8 × 10^−3^	1.55	0.807	9.0 × 10^−4^	1.37
*CALCOCO2*	0.649	0.1647	0.61	0.671	0.1103	−0.60	0.511	0.9042	0.01
*DUSP3*	0.622	0.254	−0.88	0.662	0.13	−0.94	0.642	0.1233	−0.91
*KLF2*	0.613	0.2902	−1.30	0.684	0.0852	−1.19	0.649	0.1067	−1.61

Biomarkers exceeding an AUROC threshold of 0.8 and demonstrating a fold change >2 in at least one comparison are highlighted.

#### 3.3.2 Exploratory analysis of discriminatory performance of low-performing biomarkers and expression variability in *KLF2*

Three biomarkers, *CALCOCO2, DUSP3*, and *KLF2*, did not meet the minimum diagnostic performance threshold (AUROC < 0.7), as shown in Table 2 and Supplementary Table S3.2. Due to limited discriminatory capacity, *CALCOCO2* and *DUSP3* were excluded from further analysis. Although *KLF2* also demonstrated suboptimal performance overall (PTB: LogFC = −1.30, AUROC = 0.613; EPTB: LogFC = −1.91, AUROC = 0.684; ATB: LogFC = −1.61, AUROC = 0.649), further inspection of relative quantification data revealed two distinct expression clusters within both PTB and EPTB groups (Supplementary Figure S4.5). Stratification into *KLF*2^High^ and *KLF*2^Low^ subgroups yielded markedly different diagnostic metrics:

*KLF*2^High^:

PTB: Sensitivity = 100%, Specificity = 53.3%, *p* = 8.14 × 10^−2^, AUROC = 0.725

EPTB: Sensitivity = 100%, Specificity = 53.3%, *p* = 1.096 × 10^−1^, AUROC = 0.767

ATB: Sensitivity = 83.3%, Specificity = 53.3%, *p* = 3.59 × 10^−2^, AUROC = 0.739

*KLF*2^Low^:

PTB: Sensitivity = 100%, Specificity = 100%, *p* < 1.0 × 10^−^4, AUROC = 1.000

EPTB: Sensitivity = 81.8%, Specificity = 80.0%, *p* = 2.8 × 10^−3^, AUROC = 0.849

ATB: Sensitivity = 88.89%, Specificity = 80.0%, *p* < 1.0 × 10^−4^, AUROC = 0.9074

From this study, these observations suggest that while *KLF2* is not a reliable general biomarker for tuberculosis diagnosis, its expression variability may reflect underlying biological heterogeneity, potentially linked to disease stage or host immune phenotype. The *KLF*2^Low^ subgroup demonstrated strong discriminatory power and may correspond to the configuration represented in the Sweeney3 signature algorithm. Therefore, *KLF2* may hold value for patient stratification, warranting further investigation into its mechanistic role and clinical utility in a larger patient cohort.

#### 3.3.3 Identification of best-performing biomarker combinations for discriminating ATB from controls using random forest modeling

To identify the most effective biomarker combinations for distinguishing ATB from CNTRL, random forest classification was applied using the full panel of candidate biomarkers. The model successfully classified CNTRL, PTB, and EPTB groups, achieving an OOB error rate of 0.156 (Supplementary Figure S2.6A). The top-ranking features contributing to group separation were *BATF2, GBP1*, and *SAMD9L* (Supplementary Figure S2.6B). When classification was refined to distinguish combined ATB cases from CNTRL, the model yielded a lower OOB error rate of 0.0889. In this context, *BATF2, GBP1, IFIT3*, and *SAMD9L* were identified as the most informative features (Supplementary Figures S2.7A, B).

Performance analysis of individual biomarkers revealed that *BATF2* was the strongest discriminator for PTB, but its performance was reduced in EPTB. *GBP1* and *SAMD9*L also showed strong classification ability in PTB, with slightly diminished performance in EPTB. In contrast, *IFIT3* demonstrated consistent performance across both disease forms. These findings suggest that while individual biomarkers may offer diagnostic value, they are unlikely to meet the robustness required for clinical implementation. Instead, carefully selected combinations of biomarkers may provide superior diagnostic accuracy and better alignment with the WHO's TPPs for TB diagnostics, as supported by previous studies.

### 3.4 Evaluation of INDUK biomarkers in combined diagnostic signatures

**Figure 5:**
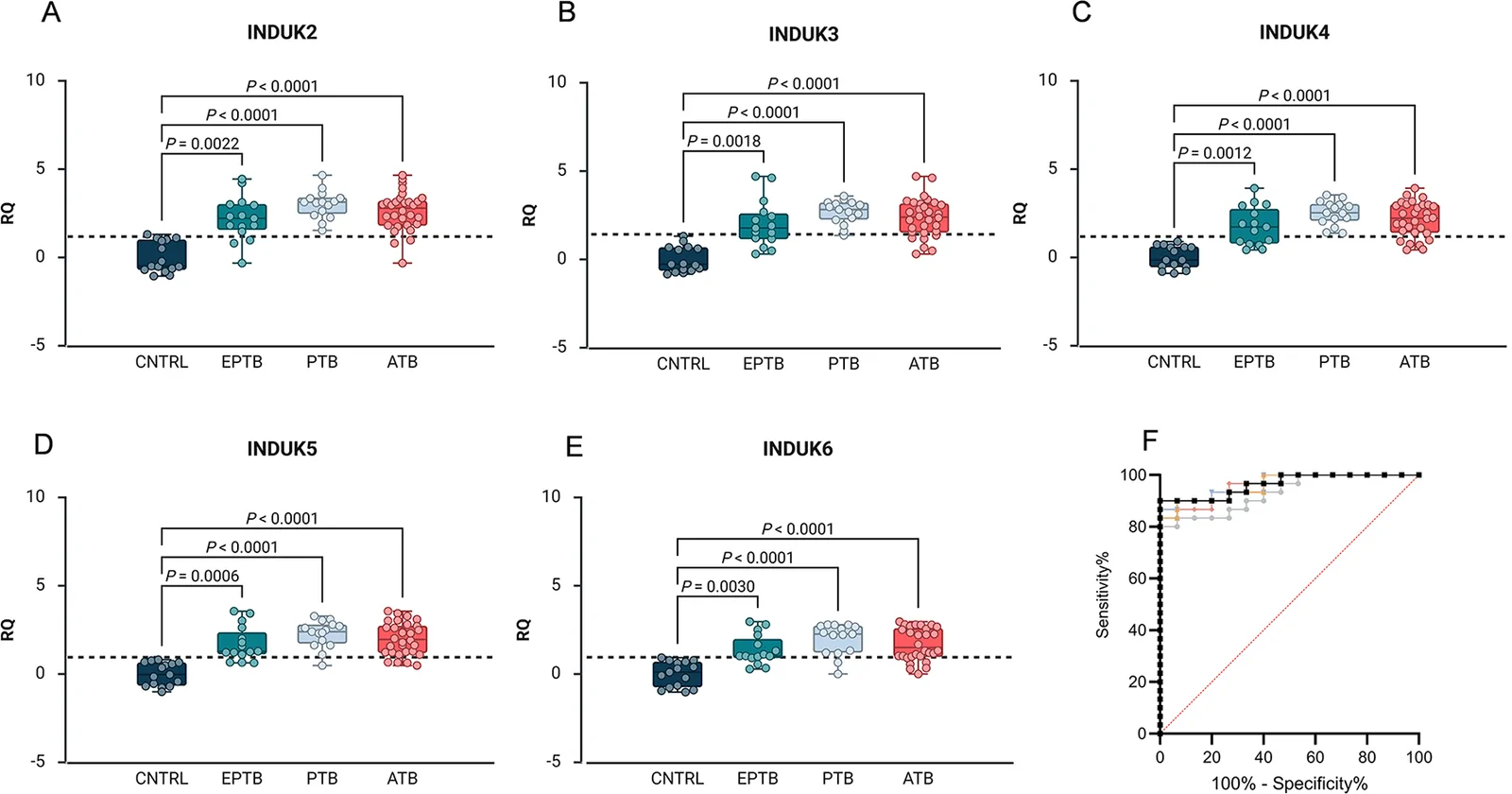
Performance of INDUK transcriptional signatures in discriminating PTB, EPTB, and ATB from CNTRL. Signature scores were calculated from relative quantification (RQ) values obtained using duplex RT-qPCR assays. Box plots show signature scores for **(A)** INDUK2, **(B)** INDUK3, **(C)** INDUK4, **(D)** INDUK5, and **(E)** INDUK6 across study groups. **(F)** Receiver operating characteristic (ROC) curves summarize the diagnostic performance of all INDUK signatures represented as 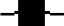 INDUK2, 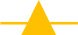 INDUK3, 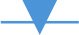 INDUK4, 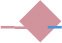 INDUK5 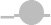 INDUK6. Created in biorender.com.

To assess the diagnostic potential of INDUK-specific biomarker signatures for identifying both PTB and EPTB, combinations of the best-performing biomarkers were evaluated using receiver operating characteristic (ROC) analysis (Figure 5).

The following diagnostic signatures were tested:

*INDUK2: GBP1* + *IFIT3*

*INDUK3: GBP1* + *IFIT3* + *SAMD9L*

*INDUK4: GBP1* + *IFIT3* + *IFITM3* + *SAMD9L*

*INDUK5: GBP1* + *IFIT3* + *IFITM3* + *SAMD9L* + *SNX10*

*INDUK6: CALCOCO2* + *GBP1* + *IFIT3* + *IFITM3* + *SAMD9L* + *SNX10*

Several combinations demonstrated excellent diagnostic performance for both PTB and EPTB, as detailed in Supplementary Table S3.3 and Supplementary Figures S4.6, S4.7. The most effective panels were INDUK2 and INDUK3. For INDUK2 (Figure 5A): PTB was discriminated from CNTRL with AUROC = 1.0 (*p* < 1.0 × 10^−4^), EPTB was discriminated from CNTRL with AUROC = 0.92889 (*p* < 1.0 × 10^−4^), ATB was discriminated from CNTRL with AUROC = 0.96444 (*p* < 1.0 × 10^−4^). For INDUK3 (Figure 5B): PTB was discriminated from CNTRL with an AUROC = 1.0 (*p* < 1.0 × 10^−4^), EPTB was discriminated from CNTRL with an AUROC = 0.92 (*p* < 1.0 × 10^−4^), ATB was discriminated from CNTRL with an AUROC = 0.96 (*p* < 1.0 × 10^−4^). Addition of further biomarkers in panels INDUK4, INDUK5, and INDUK6 did not improve diagnostic performance in this study cohort. Therefore, INDUK2 and INDUK3 were prioritized for further analysis and validation.

### 3.5 Diagnostic performance of the Roe1, Roe3 and Sweeney3 biomarker panel RT-qPCR assays

The Roe1, Roe3, and Sweeney3 biomarker diagnostic signatures were evaluated for the discrimination of TB disease groups using newly designed assays (this study), both individually or in combination using ROC analysis (Supplementary Table S3.3 and Supplementary Figures S4.7, S4.8. Roe1 (*BATF2*) performed well individually (Section 3). Roe3, consisting of *BATF2* in combination with *SCARF1* and *GBP5*, also showed good performance for discriminating PTB from CNTRL with an AUROC = 0.911 (*p* < 1.0 × 10^−4^), for EPTB discrimination from CNTRL, AUROC = 0.876 (*p* = 5.0 × 10^−4^) and for ATB: AUROC = 0.933 (*p* < 1.0 × 10^−4^). The performance of the Sweeney3 signature using the published algorithm ([*GBP5*+*DUSP3*]/2) –* KLF2* did not meet the AUROC > 0.7 threshold for a good diagnostic test. For PTB: AUROC = 0.64 (*p* = 1.9 × 10^−1^), EPTB: AUROC = 0.68 (*p* = 9.3 × 10^−2^), ATB: AUROC = 0.66 (*p* = 8.3 × 10^−2^). With a revised algorithm using the absolute *KLF2* value ([*GBP5*+*DUSP3*]/2) + *|KLF2*| performance is improved for PTB and ATB, but EPTB performance remains below acceptable thresholds. For PTB: AUROC = 0.822 (*p* = 7.0 × 10^−4^), EPTB: AUROC = 0.66 (*p* = 1.41 × 10^−1^), ATB: AUROC = 0.74 (*p* = 1.1 × 10^−2^).

### 3.6 Evaluation of the RISK6 signature using qPCR-based assays

The RISK6 signature, comprising three genes upregulated in latent tuberculosis infection (LTBI) progressors (*GBP2, FCGR1B*, and *SERPING1*) and three genes downregulated in LTBI progressors (*TUBGCP6, TRMT2A*, and *SDR39U1*), was evaluated using commercially available qPCR assays, as previously described (79). For this study, upregulated and downregulated genes were duplexed and the RISK6 score calculated from Cq values using the published algorithm. ROC analysis demonstrated good discriminatory performance of the RISK6 signature in distinguishing TB disease groups from CNTRL (Supplementary Table S3.3), with the following results: PTB: AUROC = 0.881 (*p* = 5.0 × 10^−4^) EPTB: AUROC = 0.8095 (*p* = 4.6 × 10^−4^) ATB: AUROC = 0.8452 (*p* = 3.0 × 10^−4^). These findings support the utility of the RISK6 signature as a qPCR-based diagnostic tool for active TB, with consistent performance across both pulmonary and extrapulmonary disease forms.

### 3.7 Comparative assessment of INDUK biomarker signatures and benchmarking against existing signatures and WHO TPP criteria

**Figure 6:**
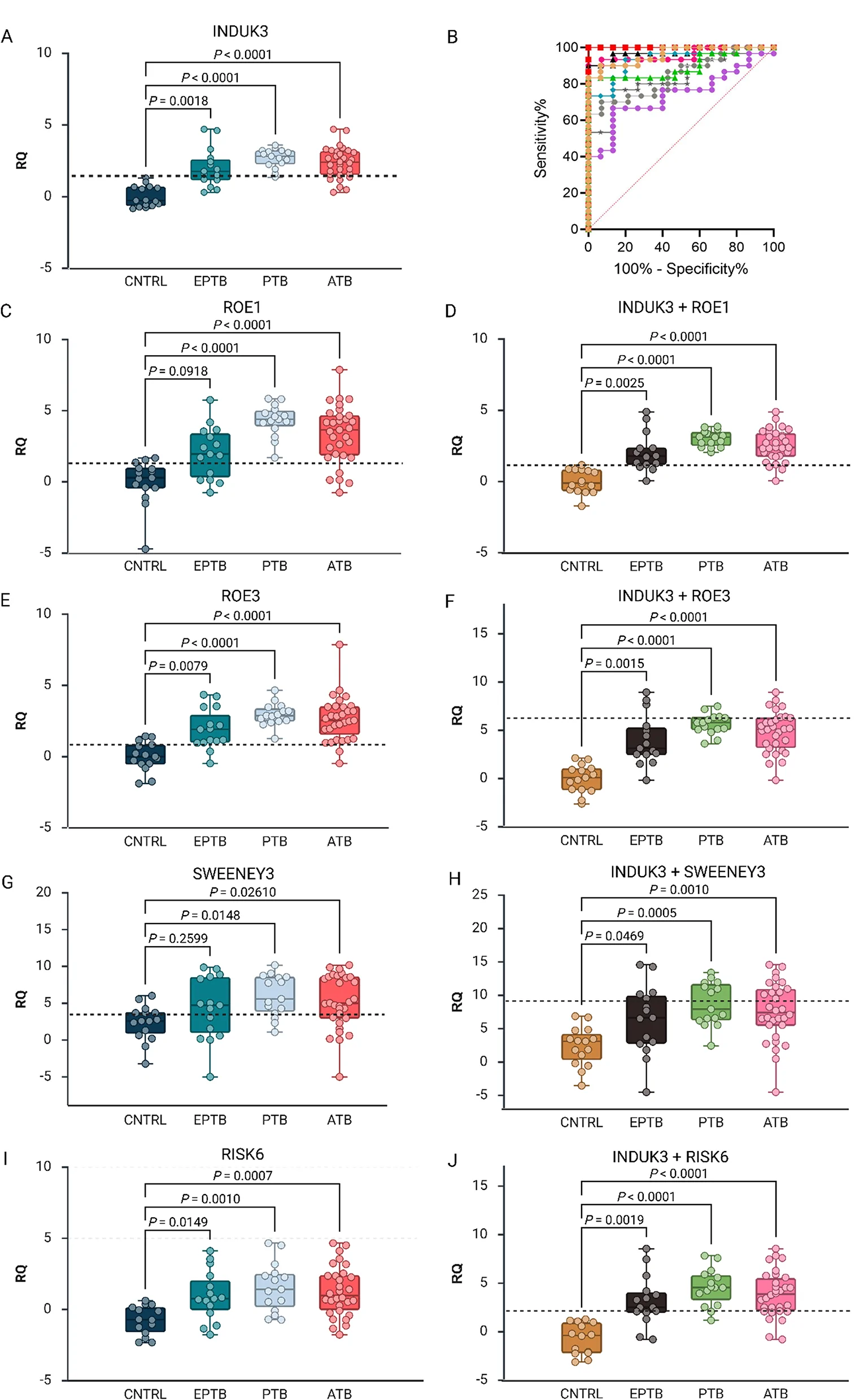
Comparison of biomarker signature performance with unified biomarker signature performance **(A)** INDUK3 **(B)** ROC curves for ATB vs control 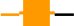 INDUK3, 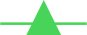 Roe1, 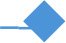 Roe3, 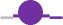 Sweeney3, 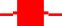 Risk6, 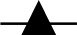 INDUK3+Roe1, 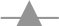 INDUK3+ Roe3, 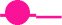 INDUK3+Sweeney3, 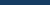 INDUK3+Risk6, **(C)** Roe1, **(D)** INDUK3+Roe3, **(E)** Roe3, **(F)** INDUK3+Roe3, **(G)** SWEENEY3, **(H)** INDUK3+SWEENEY, **(I)** RISK6, and **(J)** INDUK3+RISK6. Created in biorender.com.

To evaluate the diagnostic utility of the INDUK biomarker signatures, we compared their performance with established signatures: Roe1, Roe3, Sweeney3, and RISK6. ROC curve analyses were conducted for each signature individually and in combination (Supplementary Table S3.3; Figures 5, 6). Full ROC plots are provided in Supplementary Figures S4.1–S4.8. All signatures demonstrated strong performance for PTB, with AUROC values ranging from 0.881 to 1.0. Notably, INDUK2 and INDUK3 showed excellent diagnostic accuracy for both PTB and EPTB, with improved resolution between TB-infected individuals and healthy controls, comparable to Roe1 and Roe3. In contrast, Sweeney3 and RISK6 did not meet the minimum WHO TPP requirements for a biomarker-based diagnostic test.

#### 3.7.1 Combination signature performance

To explore whether combining biomarkers from different signatures could enhance diagnostic performance, we evaluated several hybrid signatures as presented in Supplementary Table S3.3 and Figure 6. These combinations yielded superior performance across all disease groups, particularly for EPTB, which is traditionally more challenging to diagnose. The best-performing signatures are summarized in Table 4. All signatures and combinations were assessed against the following WHO TPP criteria for confirmatory and triage tests. For confirmatory test criteria (TPP 2014 and 2024), sensitivity was assessed at the threshold yielding ≥98% specificity. For triage test criteria, specificity was recorded at 95% sensitivity (optimal), 90% sensitivity (minimal), or best achievable sensitivity/specificity if neither threshold was met.

**Table 4:** Diagnostic performance of biomarker signatures and composite biomarker signatures for distinguishing PTB, EPTB, and ATB from CNTRL, as assessed by ROC analysis and assessment against WHO TPP criteria for triage and confirmatory tests.

**Signature**	**AUROC**	**SE**	**95% CI**	***P*-value**	**Triage TPP met?**	**Confirmatory test TPP met?**	**Confirmatory update TPP met?**
**INDUK3**							
CNTRL vs. ATB	0.96	0.02517	0.911–1.00	< 0.0001	**Minimal**	**Minimal**	**Near POC**
CNTRL vs. EPTB	0.92	0.04792	0.826–1.00	< 0.0001	No	**Minimal**	No
CNTRL vs. PTB	1	0	1.00-1.00	< 0.0001	**Optimal**	**Optimal**	**Optimal**
**INDUK3**+**RISK6**							
CNTRL vs. ATB	0.9595	0.02847	0.904–1.00	< 0.0001	**Minimal**	**Minimal**	**Low complexity**
CNTRL vs. EPTB	0.9238	0.05442	0.817–1.00	0.0001	No	**Optimal**	**Low complexity**
CNTRL vs. PTB	0.9952	0.007866	0.980–1.00	< 0.0001	**Optimal**	**Minimal**	**Optimal**
**INDUK3**+**Roe1**							
CNTRL vs. ATB	0.98	0.0166	0.948–1.00	< 0.0001	**Optimal**	**Minimal**	**Low complexity**
CNTRL vs. EPTB	0.96	0.03226	0.897–1.00	< 0.0001	**Minimal**	**Optimal**	**Near POC**
CNTRL vs. PTB	1	0	1.00–1.00	< 0.0001	**Optimal**	**Optimal**	**Optimal**
**INDUK3**+**Roe3**							
CNTRL vs. ATB	0.97111	0.02263	0.927–1.00	< 0.0001	**Optimal**	**Minimal**	**Low complexity**
CNTRL vs. EPTB	0.94222	0.04391	0.856–1.00	< 0.0001	No	**Optimal**	**Near POC**
CNTRL vs. PTB	1	0	1.00–1.00	< 0.0001	**Optimal**	**Optimal**	**Optimal**
**INDUK3**+**SWEENEY3**							
CNTRL vs. ATB	0.82889	0.06083	0.710–0.948	0.0004	No	No	No
CNTRL vs. EPTB	0.7378	0.09432	0.552–0.923	0.0265	No	No	No
CNTRL vs. PTB	0.92	0.05017	0.821–1.00	< 0.0001	**Minimal**	No	No
**RISK6**							
CNTRL vs. ATB	0.8452	0.05707	0.733–0.957	0.0003	No	No	No
CNTRL vs. EPTB	0.8095	0.08313	0.646–0.972	0.0046	No	No	No
CNTRL vs. PTB	0.881	0.06219	0.759–1.00	0.0005	No	**Minimal**	**POC**
**Roe3**							
CNTRL vs. ATB	0.93333	0.03552	0.864–1.00	< 0.0001	**Minimal**	**Minimal**	**Near POC**
CNTRL vs. EPTB	0.87556	0.06336	0.751–0.999	0.0005	No	No	No
CNTRL vs. PTB	0.99111	0.01183	0.968–1.00	< 0.0001	**Optimal**	**Minimal**	**Low complexity**
**SWEENEY** +**/-** ***KLF2***							
CNTRL vs. ATB	0.74	0.07319	0.597–0.884	0.0093	No	No	No
CNTRL vs. EPTB	0.6578	0.106	0.450–0.866	0.1409	No	No	No
CNTRL vs. PTB	0.8222	0.07704	0.671–0.973	0.0026	No	No	No
**SWEENEY3**							
CNTRL vs. ATB	0.66	0.08137	0.501–0.820	0.083	No	No	No
CNTRL vs. EPTB	0.68	0.1019	0.481–0.880	0.093	No	No	No
CNTRL vs. PTB	0.64	0.1029	0.438–0.842	0.1914	No	No	No

Bold text indicates biomarker signatures that meet predefined TPP criteria, including optimal or minimal requirements for triage and/or confirmatory testing.

Seven of the 15 signatures evaluated met the optimal requirements of the TPP 2014 confirmatory test and a further 5 out of 15 met minimal requirements for discrimination of PTB from controls. For the discrimination of EPTB from controls at the reduced sensitivity threshold (80%), 7 out of 15 signatures met optimal requirements and 3 out of 15 met minimal requirements. For discrimination of ATB from controls, 10 out of 15 signatures evaluated met minimal requirements. No biomarker signature evaluated met the optimal requirements. INDUK combinations 2, 3, and 4 were able to meet the optimal sensitivity of 98% for PTB. For EPTB, INDUK5 and INDUK2 met the optimal requirements of 80% sensitivity, with INDUK5 ranking highest with an AUROC of 0.951. For ATB, INDUK2 showed the best diagnostic performance with an AUROC of 0.964 and sensitivity of 93.33% and 100% specificity, meeting the minimal TPP requirements. Overall, the combination of INDUK3 + Roe1 achieved the best performance in all comparisons.

For assessment against the TPP 2014 triage test for ruling out TB and/or identifying patients who require further investigation: 9 out of 15 signatures evaluated met optimal requirements and 1 out of 15 met minimal requirements for discrimination of PTB from controls. Two of 15 met minimal requirements for discrimination of EPTB from controls. For discrimination of ATB from controls: 2 out of 15 signatures met optimal requirements and 8 out of 15 met minimal requirements. INDUK2, 3, and 4 were able to meet the optimal TPP criteria for a triage test for PTB. INDUK5 met the minimal criteria for PTB and EPTB. No other INDUK signature met the requirements for EPTB. For ATB, only minimal TPP requirements were met for a selection of INDUK signatures.

For the revised TPP 2024 update, 7 out of 15 signatures were able to meet optimal requirements for PTB. For discrimination of EPTB and ATB from controls, no signatures were able to meet the optimal requirements; however, a larger proportion were able to meet reduced requirements for a low complexity or near-POC and POC test.

The INDUK3 + Roe1 combination fulfilled the WHO 2014 and 2024 optimal confirmatory and triage test criteria for PTB, optimal confirmatory and minimal triage criteria for EPTB, and minimal confirmatory and optimal triage criteria for ATB. INDUK3+Roe3 and INDUK3 + Roe1 + [*KLF2*] also met optimal confirmatory criteria for PTB and EPTB, and optimal triage criteria for ATB, though they did not meet minimal triage criteria for EPTB.

These combinations also satisfied various WHO 2024 TPP requirements for low complexity and near POC diagnostics. Overall, INDUK3+Roe1 demonstrated the most consistent and superior performance across both PTB and EPTB groups.

### 3.8 Decision curve analysis to assess clinical utility of biomarkers and biomarker signatures for TB triage

**Figure 7:**
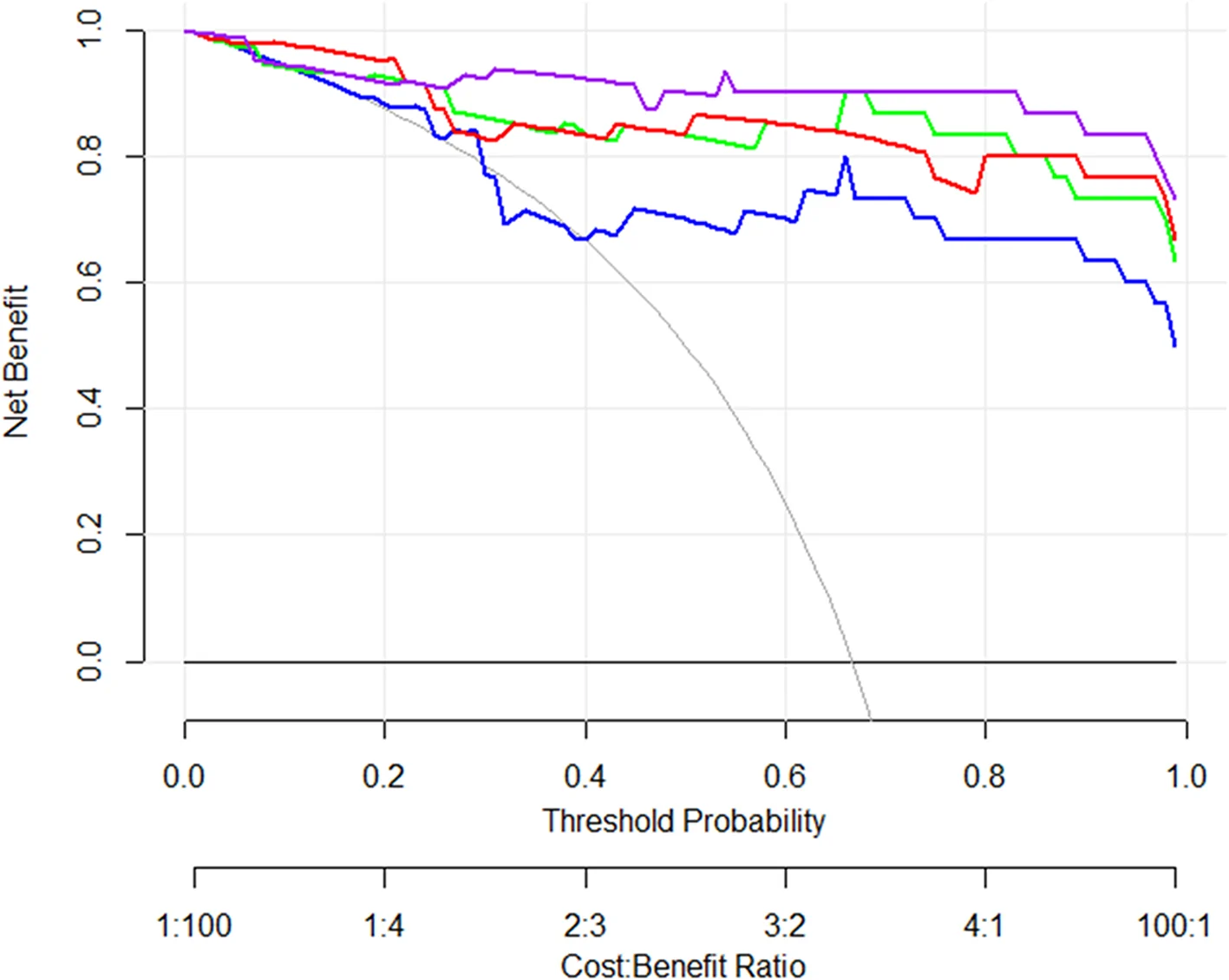
Decision curve analysis (DCA) comparing the net clinical benefit of four biomarker signatures: 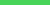 Roe1, 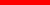 INDUK2, 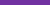 INDUK3, and —INDUK3.Roe1 —across a range of threshold probabilities. Net benefit is plotted on the y-axis, with threshold probability and corresponding cost: benefit ratios shown on the x-axes. The “treat all” (Gray curve) and “treat none” (black line) strategies are included as references.

DCA was performed to evaluate the clinical utility of four biomarker signatures for TB triage: Roe1 (*BATF2*), INDUK2 (*GBP1*+*IFIT3*), INDUK3 (*GBP1*+*IFIT3*+*SAMD9L*), and the unified signature INDUK3.Roe1 (*BATF2*+*GBP1*+*IFIT3*+ *SAMD9L*) across a range of threshold probabilities relevant to clinical decision-making (Figure 7). All signatures demonstrated greater net benefit than the “treat none” strategy, across nearly the entire range of threshold probabilities, indicating potential clinical value for ruling in TB cases. The INDUK3.Roe1 signature consistently showed the highest net benefit across most thresholds between 5% and 60%, suggesting superior performance for balancing true positive and false positive triage outcomes. Roe1 displayed good net benefit at lower thresholds (< 30%) but declined at higher thresholds, indicating it may be more suitable when the priority is to minimize missed cases, at the expense of more false positives. INDUK2 and INDUK3 had comparable performance, with net benefits generally below INDUK3.Roe1 but above *BATF2* in the mid-threshold range. These results support the use of multiplex qPCR signatures, particularly INDUK3.Roe1, as effective triage tools in high-prevalence, resource-limited settings where optimal allocation of confirmatory diagnostics is critical.

### 3.9 Evaluation of INDUK biomarker quadruplex qPCR assays

**Figure 8:**
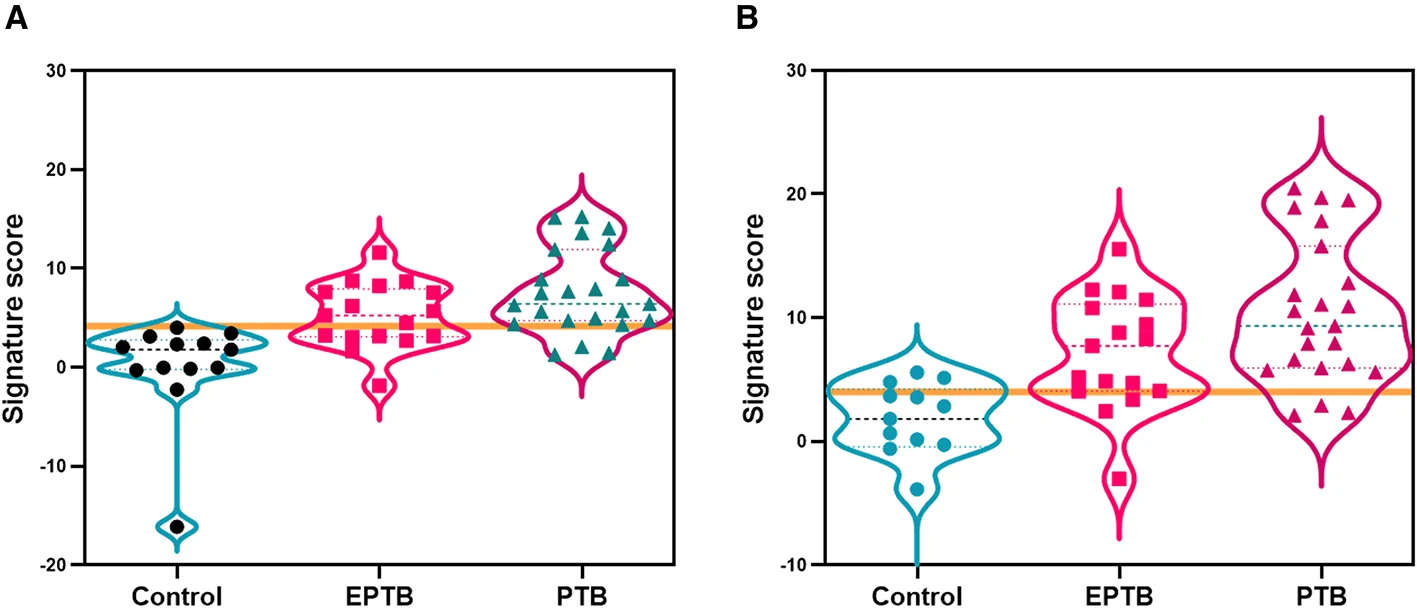
Performance of **(A)** INDUK2 and **(B)** INDUK3 signatures using quadruplex RT-qPCR assays.

To explore the translational potential of the INDUK biomarker signatures into a low-footprint diagnostic format, we developed multiplex RT-qPCR assays capable of simultaneous detection of multiple targets within a single reaction. Two quadruplex assays were designed, each incorporating three target genes and one reference gene per well: Quadruplex Assay A: *GBP1, CALCOCO2, SAMD9L* with *RPL13A* as the internal housekeeping gene; Quadruplex Assay B: *IFIT3, IFITM3, SNX10* with *PGK1* as the internal housekeeping gene. All samples were reanalyzed using these multiplex assays, and relative gene expression calculated using both *RPL13A* and *PGK1* for normalization purposes (Supplementary Table S3.4; Figure 8).

The goal was to determine whether comparable diagnostic performance could be achieved with the multiplex format relative to the previously validated duplex assays. Using established cut-off values from INDUK3 and INDUK4 evaluations, we assessed the discriminatory power of the multiplex assays by calculating PPV and NPV. At a cut-off value of 3, the INDUK2 signature yielded a PPV of 91.89% and NPV of 62.5%. At a cut-off value of 4, the INDUK3 quadruplex assay achieved a PPV of 91.89% and NPV of 62.5%. These results indicate that a positive test result above the defined threshold is highly predictive of TB disease, supporting the feasibility of multiplex qPCR assays for streamlined, high-throughput diagnostic applications.

To understand the utility of the INDUK3 assay across different epidemiological settings, a scenario analysis was conducted to estimate PPV and NPV at varying TB prevalence levels (5%, 10%, 20%, and 41%) based on a baseline PPV of 91.89% and NPV of 62.5% (Table 5). Thirty-one percent TB prevalence was set as baseline, based on estimates of current TB prevalence in India (12). This analysis helps contextualize performance in triage applications, where prevalence-driven shifts in predictive value are critical. At the baseline prevalence of 31%, PPV and NPV were 91.9% and 62.5%, respectively, consistent with the observed test performance characteristics. In low-prevalence settings (5%), the PPV was estimated at 42.4%, while the NPV remained high at 98.4%, indicating a strong ability to correctly identify true negatives but moderate ability to confirm true positives. As prevalence increased to 10% and 20%, PPV improved to 60.9% and 77.8%, respectively, while NPV declined to 96.3% and 91.0%, reflecting a greater probability of true positive results but decreasing confidence in negative results. When prevalence increased further to 41%, PPV reached 94.5%, indicating high reliability of positive test results in high-burden settings. However, NPV dropped substantially to 48.5%, highlighting a diminished capacity to confidently rule out TB among those testing negative. Overall, these results demonstrate that the diagnostic test's PPV increases with rising TB prevalence, while NPV decreases, underscoring the importance of considering local disease prevalence when interpreting test results.

**Table 5:** PPV and NPV of triage test at varying TB prevalence levels (5%, 10%, 20%, 31%, and 41%), assuming constant sensitivity (90%) and specificity (80%).

**TB prevalence**	**PPV (%)**	**NPV (%)**
5%	42.4%	98.4%
10%	60.9%	96.3%
20%	77.8%	91.0%
31% (Baseline)	91.9%	62.5%
41%	94.5%	48.5%

This scenario analysis supports assessment of test utility across different epidemiological contexts.

## 4 Discussion

The WHO has set ambitious global targets as part of the End TB Strategy, aiming for a 95% reduction in TB mortality and 90% reduction in TB incidence by 2035, relative to 2015 levels (1). A central pillar of this strategy is early, accurate diagnosis of TB across all disease presentations. However, current diagnostic tools remain limited, particularly in low-resource settings and for EPTB, where traditional sputum-based diagnostics are not appropriate. The need for accurate, cost-effective, and accessible diagnostic solutions is especially critical in high-burden countries such as India (79, 80). Early diagnosis is a cornerstone of the strategy, and host transcriptional biomarkers remain a promising avenue for development of rapid, blood-based tests (7–10). This study conducted both a reanalysis of publicly available transcriptomic datasets to assess the diagnostic performance of TB-associated biomarkers and experimental validation using qPCR to further validate these observations and evaluate novel biomarker combinations.

Previously, Sweeney's integrated meta-analysis reported high AUROCs for the Sweeney3 in case–control cohorts, often exceeding 0.85, although independent validations yielded somewhat lower values (0.75–0.85) depending on cohort composition (36). Prospective RT-qPCR evaluation in community screening demonstrated more modest discrimination (AUROCs 0.63–0.79) (37). In contrast, a multicountry evaluation of the six-gene RISK6 signature showed excellent diagnostic performance, with AUROCs of 0.94 and 0.93 for distinguishing active TB from healthy donors and latent TB, respectively, achieving sensitivity of 90.9% and specificity of 87.8%, and meeting or approaching the WHO triage test TPP (28). In the CORTIS head-to-head RT-qPCR study, all four competitor signatures (RISK6, Roe3, Roe1, and Sweeney3) performed very well for symptomatic TB, with Roe3 achieving the highest symptomatic AUROC (0.97) and RISK6 notable for its prognostic durability, while Roe1 showed particular strength in people living with HIV. However, across community-based screening of largely asymptomatic individuals, all signatures demonstrated only moderate discrimination (AUROCs 0.61–0.78), with none reliably meeting WHO triage benchmarks (37).

Mechanistically, several biomarkers (*GBP1, GBP5, IFIT3, BATF2*) are consistently identified across independent studies and are strongly associated with interferon-regulated innate immune pathways, neutrophil activation, and intracellular pathogen defense (31, 43, 81). Reanalysis of previously published datasets gave insights into early and established patterns of immune biomarker expression in TB. Dataset GSE76703 revealed early and sustained expression of several INDUK3 genes from Week 1 after infection in a NHP model, with *GBP1, IFIT3*, and *SAMD9L* exhibiting consistent upregulation. In contrast, several established biomarkers, such as those in the Sweeney3 and Roe3 panels, showed variable or limited early expression. Analysis of human datasets GSE42834 and GSE144127 confirmed preferential expression of *BATF2, GBP5, SERPING1, GBP1*, and *IFIT3* in TB compared to other diseases, including sarcoidosis, pneumonia, and malignancy. Notably, *BATF2* expression was significantly higher in PTB compared to sarcoidosis in GSE42834, supporting its diagnostic potential in clinical differentiation. However, this result was not replicated in dataset GSE144127. Random forest modeling of these datasets revealed the prominence of key biomarkers e.g., *BATF2, GBP1*; however, rank-based performance differed somewhat between the different datasets.

Our findings also suggest divergent immune activation profiles in PTB and EPTB. *BATF2*, a marker of type II IFN-γ signaling, was preferentially expressed in PTB patients and not EPTB, consistent with heightened type II interferon responses in pulmonary disease. *IFIT3*, associated with type I interferon responses, was more elevated in EPTB, suggesting more of a bias toward an IFNα/β immune activation pathway (82–92). These distinctions reinforce the importance of biomarker selection tailored to TB disease presentation. Combining markers responsive to different interferon pathways would appear to be a successful strategy for developing a universal diagnostic applicable to all TB forms. Variability in expression due to disease stage, host genetics, co-infections, and other factors underscores the need for panels that minimize redundancy and maximize robustness across populations (93–101).

We evaluated established and novel blood-based transcriptional biomarker signatures using newly designed duplex qPCR assays and a refined RT-qPCR framework (normalizing to internal housekeeping gene *PGK1)*, focusing on their ability to detect both PTB and EPTB in an exploratory, pilot study of TB patients and controls in a South Indian TB patient cohort. Our findings offer valuable insight into the potential of these biomarkers to inform the development of a high-performing, scalable, and affordable diagnostic test platform, aligned with WHO TPPs for TB diagnostics. Most biomarkers performed well, with the exception of *CALCOCO2, DUSP3*, and *SNX10*, whose investigation was discontinued. In this study, expression of *KLF*2, a component of the Sweeney3 signature, was variable (into *KLF*2^High^ and *KLF*2^Low^ subgroups) and negatively affected diagnostic performance when applied using the original algorithm, which impacted accuracy. We showed that performance could be significantly improved using the absolute rather than the relative RQ value within the formula, in our Indian cohort. Upregulation or downregulation of *KLF2* could be used to stratify patients subject to further investigation but appears to negatively impact the Sweeney3 signature in this study. Testing on a larger cohort is warranted with the prediction of *KLF2* as potentially important biomarker for stratification of TB patients, with as yet undefined contributary factors e.g., microbiome status which may contribute to disease pathology (93–95).

Various combinations of biomarkers were modeled, with good performance; however, the novel INDUK3 panel, comprising *GBP1, IFIT3*, and *SAMD9L*, demonstrated the strongest diagnostic potential in PTB and notably in EPTB cases, which are typically harder to diagnose due to limitations with sample accessibility and variable performance of other methods, including GeneXpert (27, 102–111). These combined results improved overall performance for ATB discrimination. We then compared the INDUK panels against leading diagnostic signatures: Roe1/3, Sweeney3, and RISK6, which all performed well in detecting PTB. Combining high-performing elements from multiple panels (e.g., *BATF2, GBP1, IFIT3, SAMD9L*) improved classification accuracy and supported the concept of modular diagnostic panel design, aligned with an understanding of the biological heterogeneity of TB. The INDUK3+Roe1 signature met the WHO's original TPPs for triage and confirmatory testing and aligns with the updated 2024 TPPs for molecular diagnostics suitable for near-POC use. Importantly, the design would support high sensitivity and specificity across different TB presentations. Other combined panels also showed good performance; however, these were consistently inferior to INDUK3+Roe1 for EPTB detection. This four-gene combination fulfilled the WHO's optimal TPPs for triage tests and the minimal criteria for confirmatory tests, indicating its strong potential for clinical translation.

The successful validation and progression of biomarker signatures have been impeded by variability in study design, platform and laboratory variability, heterogeneity within and between population cohorts, regulatory restrictions and costs, and the inability to reach stringent WHO TPPs. To assess real-world utility, we applied DCA to evaluate net clinical benefit across varying threshold probabilities. All evaluated signatures, Roe1, INDUK2, INDUK3, and the combined signature INDUK3+Roe1, demonstrated net benefit over “treat none” and “treat all” strategies between 5% and 60% threshold probability. These values correspond to plausible pretest probabilities in high-burden, low-resource settings and reflect the clinical uncertainty faced by frontline providers. Among all models, INDUK3+Roe1 provided the highest net benefit across most thresholds, particularly between 20% and 50%, a range highly relevant for TB triage decision-making in India. This suggests that the addition of *BATF2* (Roe1) could improve real-world clinical performance, allowing better differentiation of true TB cases while minimizing unnecessary follow-up of true negatives. While the Roe1 (*BATF2*) signature alone showed good net benefit at lower thresholds, which is ideal where the goal is high sensitivity and low false negatives, it underperformed at higher thresholds. This makes it less suitable where specificity is a priority or confirmatory resources are scarce. In contrast, the INDUK3-based panels offered more consistent performance across a broad range of clinical scenarios, striking a better balance between ruling in and ruling out TB.

We have demonstrated that these assays could be configured in multiplex format, with comparable results, offering the potential for minimal format assay access. They offer many advantages with respect to the development of a multiplex qPCR platform for TB diagnosis, platform flexibility, and potential for integration into current molecular laboratory workflows without the need for new hardware, and thus, they improve cost-effectiveness. The RT-qPCR format used in this study would be particularly suited to hospital or regional microscopy laboratory settings where PCR instruments are commonly found. It is hardware-agnostic, allowing deployment on existing qPCR systems already installed in many diagnostic laboratories. It could offer a lower cost per test compared to platforms like GeneXpert (~$77.9/test) (114), with our estimated cost being < $10 per assay, excluding RNA extraction. Unlike GeneXpert, our approach does not require cartridges, which significantly reduces recurring costs and dependency on proprietary consumables. The use of blood-based sampling would improve feasibility in outpatient and primary care settings and eliminate the need to collect sputum or perform invasive biopsies, which is especially beneficial for diagnosing EPTB (112).

In conclusion, this exploratory pilot study demonstrates that the composite INDUK3+Roe1 biomarker panel, delivered via multiplex RT-qPCR, could offer strong diagnostic accuracy for both PTB and EPTB, meeting critical WHO diagnostic benchmarks. The assay's potential accessibility, compatibility with existing platforms, and affordability would make it highly attractive for implementation in high-TB-burden settings such as India. Assessment of integration into clinical workflows, with further optimization of blood collection and RNA processing methods and further evaluation of potential for TB disease stratification based on differential interferon response markers is required. Future work will focus on further large-scale, multicenter validation studies to confirm diagnostic performance across diverse populations and disease comparators. Our findings support the feasibility of developing a “one-size-fits-all” blood-based diagnostic tool for TB that is accurate, scalable, and aligned with global public health priorities.

## 5 Limitations of the study

This study has several important limitations. First, the small cohort size restricts the generalizability of the findings and may contribute to an overestimation of diagnostic accuracy metrics. ROC analyses were conducted on the entire dataset without division into independent training and testing cohorts, due to limited sample size. Consequently, the diagnostic accuracy results for biomarker signatures should be interpreted with caution and considered preliminary. Larger, demographically diverse population studies are required to allow robust model training, independent validation, and assessment of diagnostic reproducibility. Second, the lack of comparison with other respiratory and infectious diseases, particularly non-tuberculous mycobacterial (NTM) infections, may result in an overestimation of specificity. Inclusion of clinically relevant differential diagnoses such as pneumonia and NTMs are currently being addressed in an expanded validation study involving over 45 participants per group. Finally, LTBI status was not confirmed in control participants using IGRAs, raising the possibility that undiagnosed LTBI cases were inadvertently included. In the ongoing follow-up study, all control participants will undergo IGRA testing to enable stratification into IGRA-positive and IGRA-negative subgroups, thereby improving accuracy in biomarker evaluation.

## Data Availability

The original contributions presented in the study are publicly available. This data can be found here: https://doi.org/10.6084/m9.figshare.31073812.

## Footnotes

1
https://www.quantumdx.com/products-solutions/platforms-assays/tb-hira/

## Supplementary material

Supplementary Table S1.1 All information and diagnosis assessment criteria for patients and controls used in the study.

Supplementary Table S1.2 All primer and probe sequences, dyes, and quenchers used in the study.

Supplementary Table S1.3 All hydrolysis probe fluorophore labels substitutions from FAM to Texas Red or Cy5 used in the quadruplex assays.

Supplementary Table S1.4 All study biomarker STRING version 12.0 functional enrichment data.

Supplementary Table S1.5 All study biomarker STRING version 12.0 functional enrichment identified processes.

Supplementary Table S1.6 All study biomarker STRING version 12.0 K means clustering data and descriptions.

Supplementary Table S1.7 ANOVA corrected and uncorrected p and fold change values from GeneSpring 14.9 analysis of NHP expression dataset GSE76703.

Supplementary Table S1.8 ANOVA corrected and uncorrected p and fold-change values from GeneSpring 14.9 analysis of human expression dataset GSE42834.

Supplementary Table S1.9 ANOVA corrected and uncorrected p and fold-change values from GeneSpring 14.9 analysis of human expression dataset GSE144127.

Supplementary Figure S2.1 Random forest model showing **(A)** classification of NHP data set GS76073 and **(B)** biomarkers of greatest importance for modeling RF classification of NHP data set GS76073.

Supplementary Figure S2.2 Graphic depiction of AUROC values for individual biomarkers discriminating Week 1-Week 6 after *M. tuberculosis* challenge from pre-bleed samples in a NHP model of TB infection indicating variation of diagnostic performance with time.

Supplementary Figure S2.3 Random forest model showing **(A)** classification of Human data set GSE42834 and **(B)** biomarkers of greatest importance.

Supplementary Figure S2.4 Random forest model showing **(A)** classification of human dataset GSE144127 and **(B)** biomarkers of greatest importance.

Supplementary Figure S2.5 Heatmap showing biomarker expression of relative quantification from duplex RT-qPCR assays.

Supplementary Figure S2.6 Random forest modeling of RQ data from RT-qPCR assays showing classification of **(A)** CNTRL, EPTB, and PTB with **(B)** biomarker features of greatest importance.

Supplementary Figure S2.7 Random forest modeling of RQ data from RT-qPCR assays showing classification of **(A)** classification of CNTRL and ATB with **(B)** biomarkers of greatest importance.

Supplementary Table S2.1 Comparison of AUROC values for individual biomarkers discriminating Week 1-Week 6 following *M. tuberculosis* challenge from pre-bleed samples in a NHP model of TB infection indicating variation of diagnostic performance with time.

Supplementary Table S3.1 Differential expression of selected biomarkers across tuberculosis disease states. Log_2_ fold changes in gene expression are shown for EPTB, PTB, and ATB relative to CNTRL. Positive values indicate upregulation, and negative values indicate downregulation compared with controls.

Supplementary Table S3.2 Diagnostic performance of individual biomarkers for discrimination between healthy controls and tuberculosis disease groups. Receiver operating characteristic (ROC) analysis was performed for each biomarker to distinguish CNTRL from ATB, EPTB, and PTB. The table reports the area under the ROC curve (AUROC) with standard error, 95% confidence interval, and corresponding P value. Optimal cut-off values were determined based on ROC analysis. Sensitivity and specificity are presented as percentages with 95% confidence intervals, along with likelihood ratios where applicable. Biomarkers labelled as *KLF2* (HIGH) and *KLF2* (LOW) represent stratified expression thresholds for *KLF2* evaluated separately.

Supplementary Table S3.3 Diagnostic accuracy of multi-gene transcriptional signatures for tuberculosis across WHO TPP criteria. ROC analyses performed for individual and composite transcriptional signatures to discriminate CNTRL from PTB, EPTB, and ATB. For each signature, AUROC with standard error, 95% confidence interval (CI), P value, t-test significance, and overall log_2_ fold change are reported. Optimal cut-off values were derived from ROC analysis, and corresponding sensitivity, specificity, 95% CIs, likelihood ratios (LR+), and Youden index are shown. Signature performance was further evaluated against WHO TPP benchmarks for: (i) 2014 confirmatory tests, (ii) 2014 triage tests, and (iii) the 2024 updated TPP, including optimal, minimal, low-complexity, near point-of-care (POC), and POC use cases. For each TPP category, sensitivity and specificity thresholds were assessed, and signatures were annotated as “Met” or “Not met” based on whether they satisfied the corresponding criteria. Composite signatures are defined as arithmetic means or combinations of normalized gene expression values as indicated.

Supplementary Table S3.4 Diagnostic performance of multiplex transcriptional signatures measured by a 4-plex assay for discrimination between healthy controls and tuberculosis disease groups. ROC analysis performed for individual multiplex gene expression signatures measured using a quadruplex assay to distinguish CNTRL from ATB, PTB, and EPTB. For each comparison, AUROC with standard error, 95% confidence interval (CI), P value, and the optimal diagnostic cut-off derived from ROC analysis are reported. Sensitivity and specificity are presented with 95% CIs, along with normalized sensitivity and specificity and the Youden Index as a measure of overall diagnostic performance.

Supplementary Figure S4.1 Graphical display of duplex qPCR RQ values for *GBP1, IFIT3*, and *SAMD9L* and associated ROC curve plots.

Supplementary Figure S4.2 Graphical display of duplex qPCR RQ values for *CALCOCO2, IFITM3*, and SNX10 and associated ROC curve plots.

Supplementary Figure S4.3 Graphical display of duplex qPCR RQ values for *BATF2, SCARF1*, and *GBP5* and associated ROC curve plots.

Supplementary Figure S4.4 Graphical display of duplex qPCR RQ values for *DUSP3* and associated ROC curve plots.

Supplementary Figure S4.5 Graphical display of duplex qPCR RQ values for *KLF2* combined, *KLF2* High and *KLF2* Low and associated ROC curve plots.

Supplementary Figure S4.6 Graphical display of duplex qPCR RQ values for the combined INDUK2, INDUK3, and INDUK4 signatures and associated ROC curve plots.

Supplementary Figure S4.7 Graphical display of duplex qPCR RQ values for combined INDUK5, INDUK6 signatures and the Roe3 signature and associated ROC curve plots.

Supplementary Figure S4.8 Graphical display of duplex qPCR RQ values for the Sweeney3, improved Sweeney3, and RISK6 signatures and associated ROC curve plots.

Supplementary Figure S4.9 Graphical display of duplex qPCR Rq values for INDUK3+Roe3 combined, INDUK3+Roe1 combined, INDUK3+Sweeney3 combined and associated ROC curve plots.

Supplementary Figure S4.10 Graphical display of duplex qPCR Rq values for INDUK3+RISK6 combined, BATF2, GBP1, GBP5, IFIT3, KLF2 and SAMD9L combined average and BATF2, GBP1, IFIT3, KLF2 and SAMD9L combined average and associated ROC curve plots.
