# Supplementary material for: Evaluation of host-immune biomarker signatures as multiplex qPCR diagnostic assays: a pilot study toward meeting WHO target product profiles for TB diagnosis in India
Source: Front Tuberc. 2026 Feb 16;3:1678039. doi: 10.3389/ftubr.2025.1678039 (PMC13494995; doi:10.3389/ftubr.2025.1678039)
Supplement: Supplementary file 1 [file Data_Sheet_4.pdf]

Figure S4.1

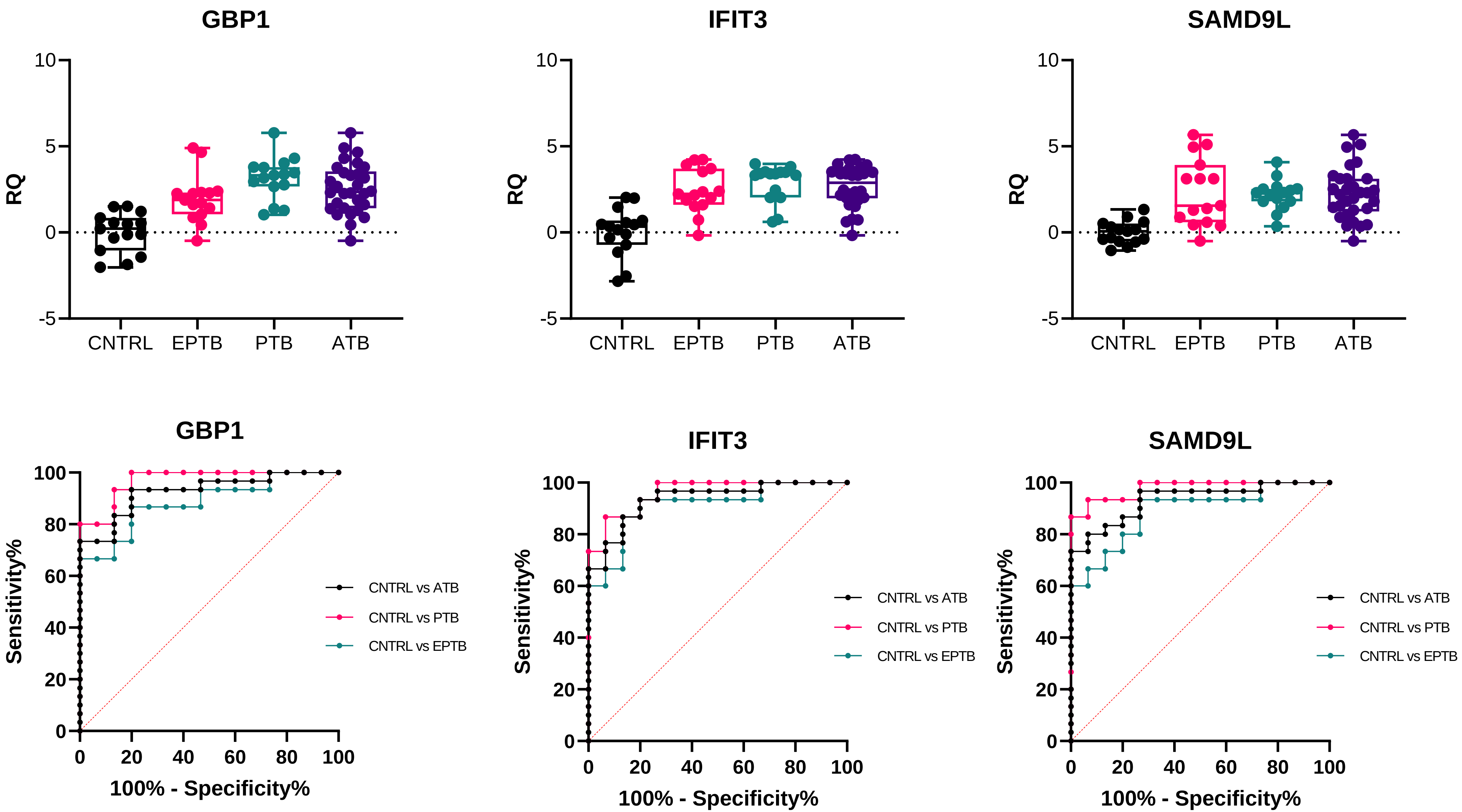

GBP1
IFIT3
SAMD9L
RQ
10
5
0
-5
CNTRL
EPTB
PTB
ATB
GBP1
IFIT3
SAMD9L
Sensitivity%
100% - Specificity%
0
20
40
60
80
100
CNTRL vs ATB
CNTRL vs PTB
CNTRL vs EPTB

Figure S4.2

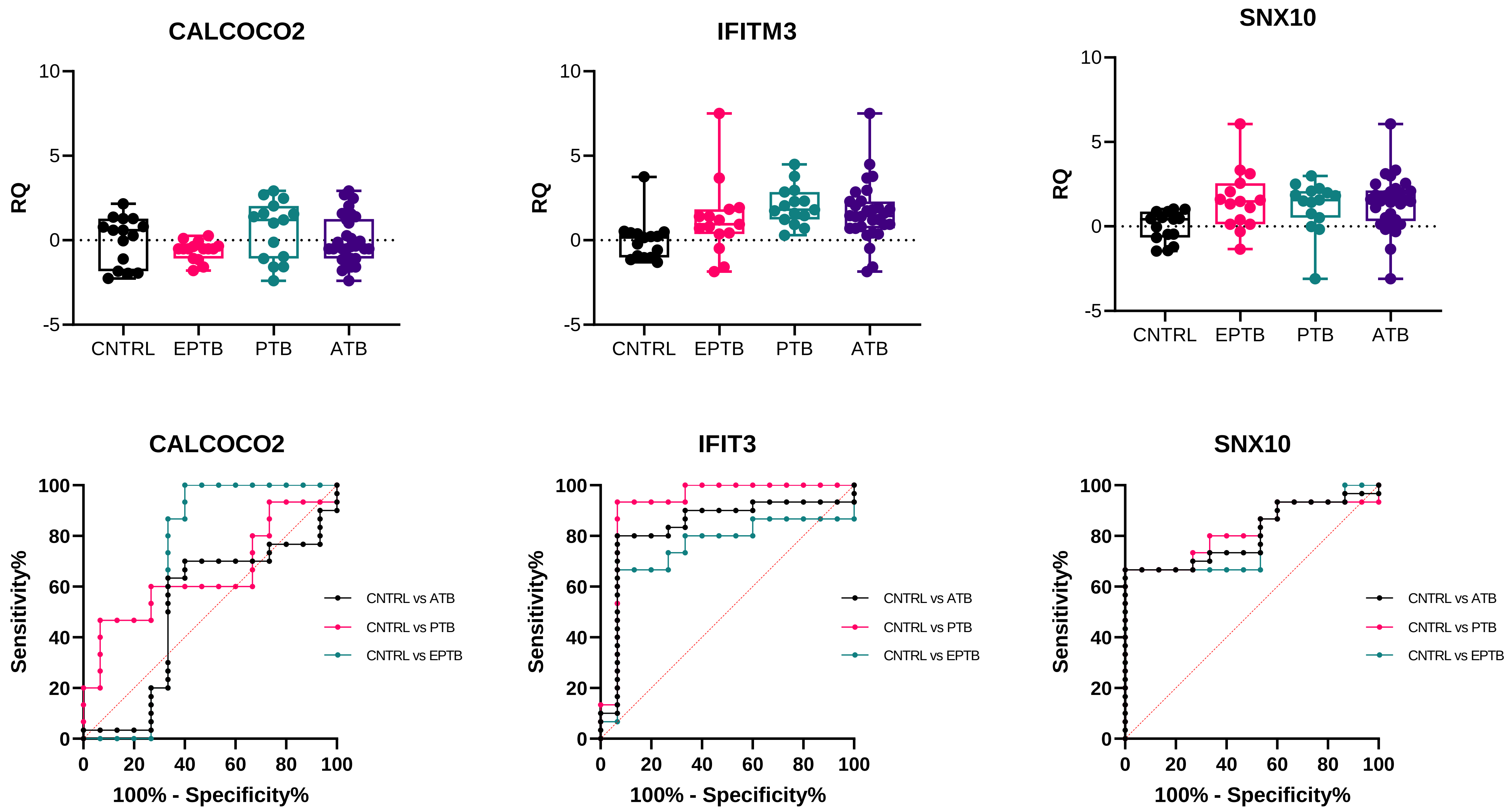
CALCOCO2
IFITM3
SNX10
RQ
10
5
0
-5
CNTRL
EPTB
PTB
ATB
CALCOCO2
IFIT3
SNX10
Sensitivity%
100% - Specificity%
0
20
40
60
80
100
CNTRL vs ATB
CNTRL vs PTB
CNTRL vs EPTB

Figure S4.3

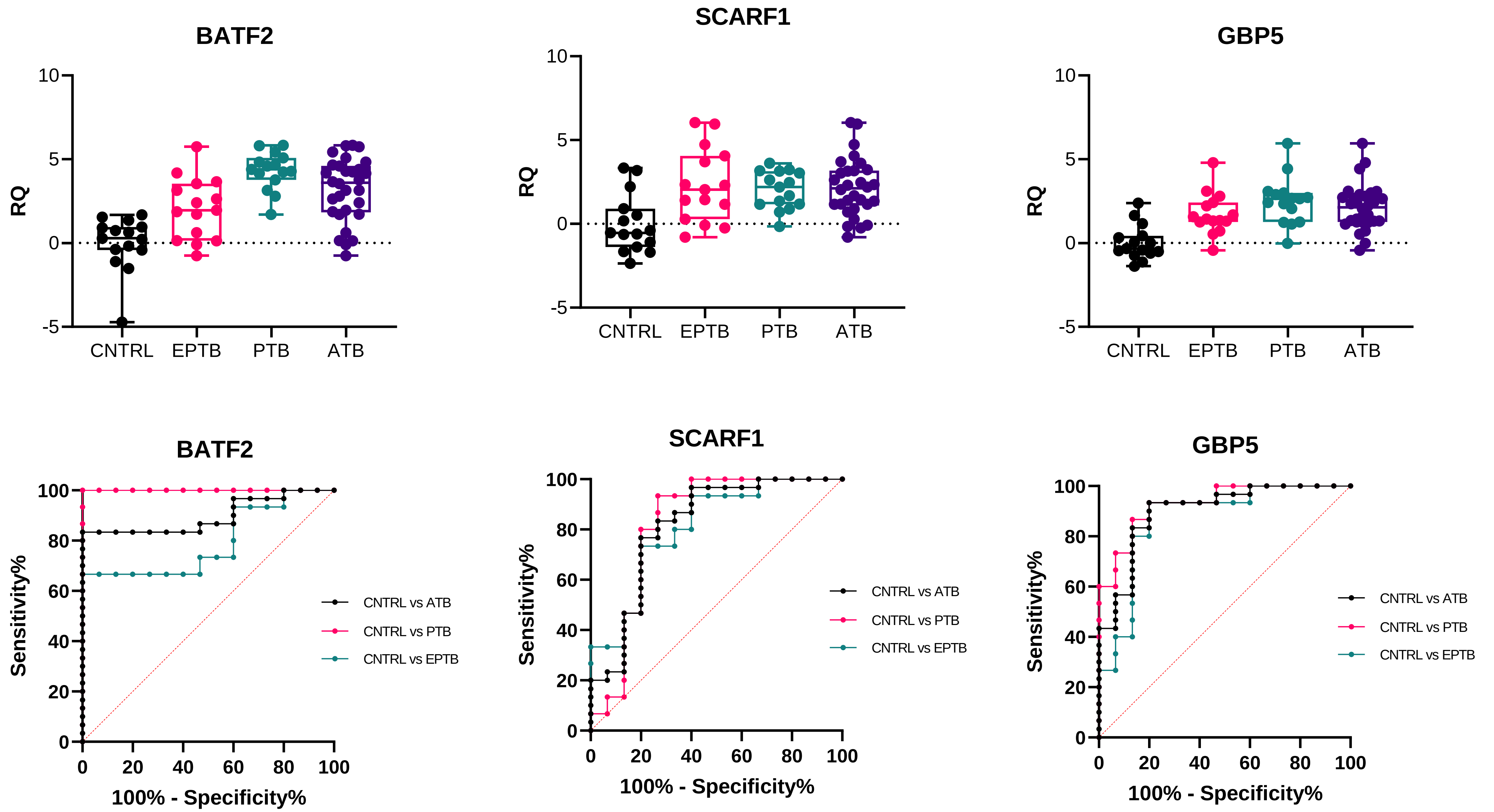

BATF2
SCARF1
GBP5
RQ
CNTRL
EPTB
PTB
ATB
Sensitivity%
100% - Specificity%
CNTRL vs ATB
CNTRL vs PTB
CNTRL vs EPTB

Figure S4.4

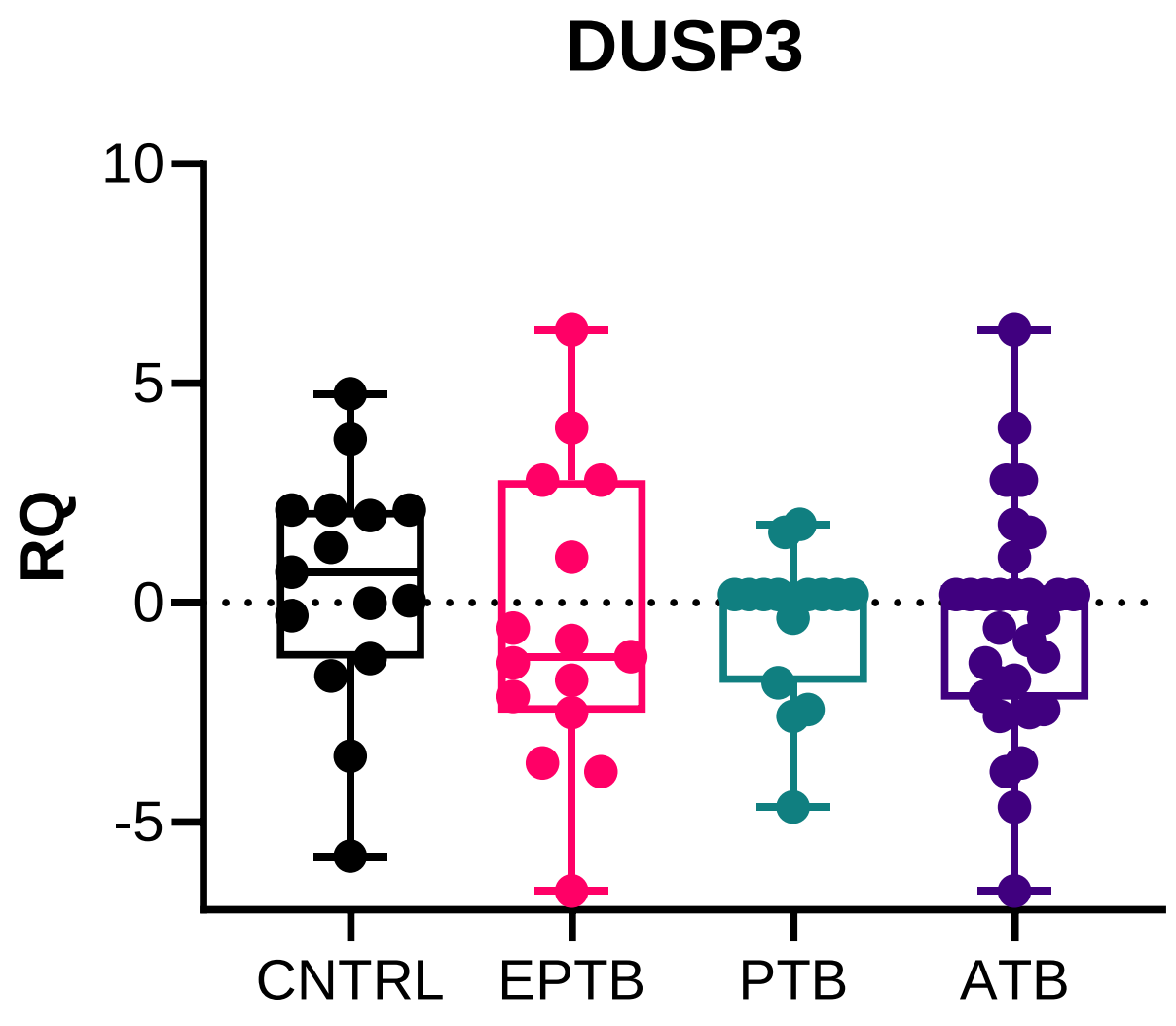
DUSP3
10
5
0
-5
RQ
CNTRL
EPTB
PTB
ATB

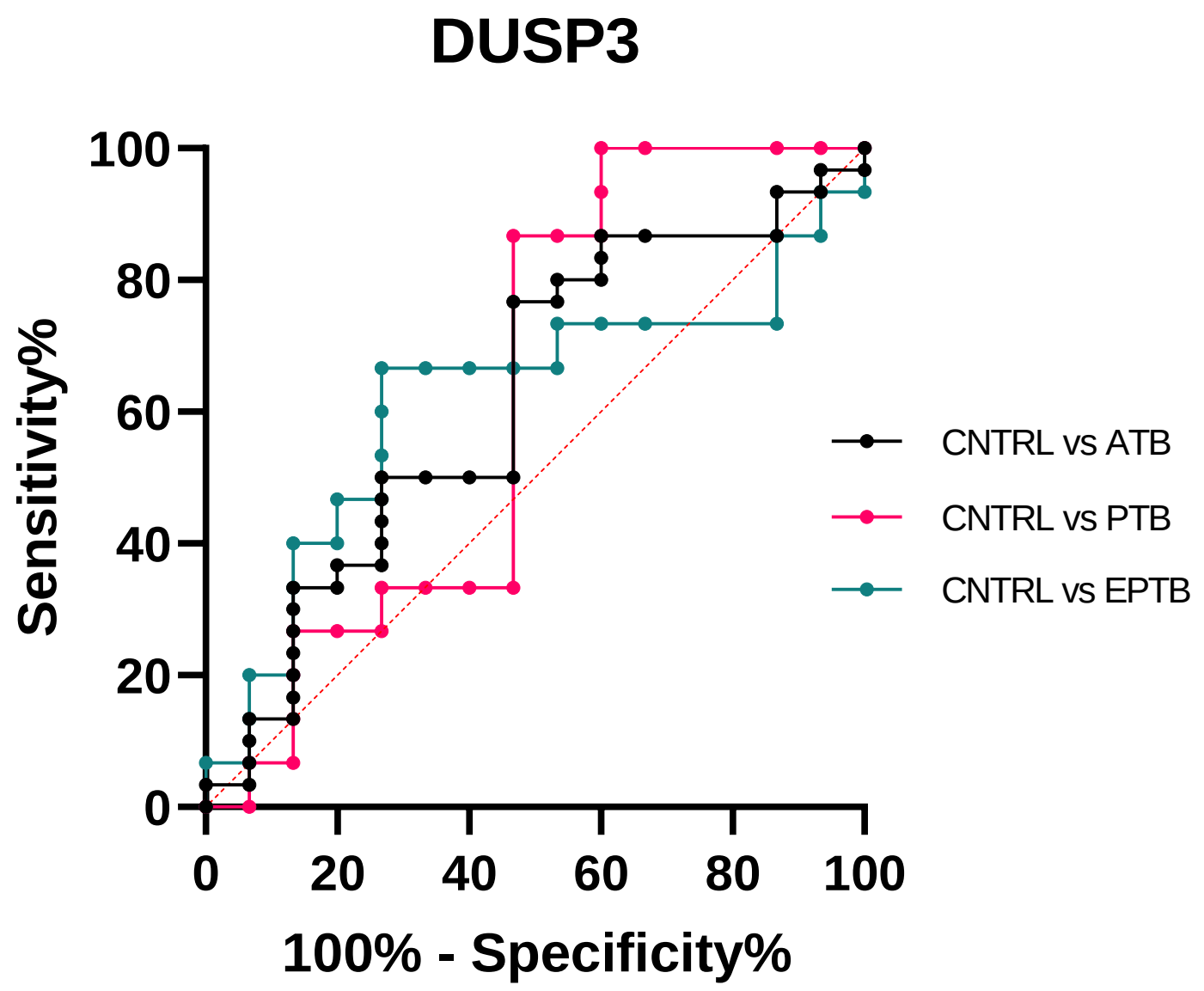
DUSP3
100
80
60
40
20
0
Sensitivity%
0
20
40
60
80
100
100% - Specificity%
CNTRL vs ATB
CNTRL vs PTB
CNTRL vs EPTB

Figure S4.5

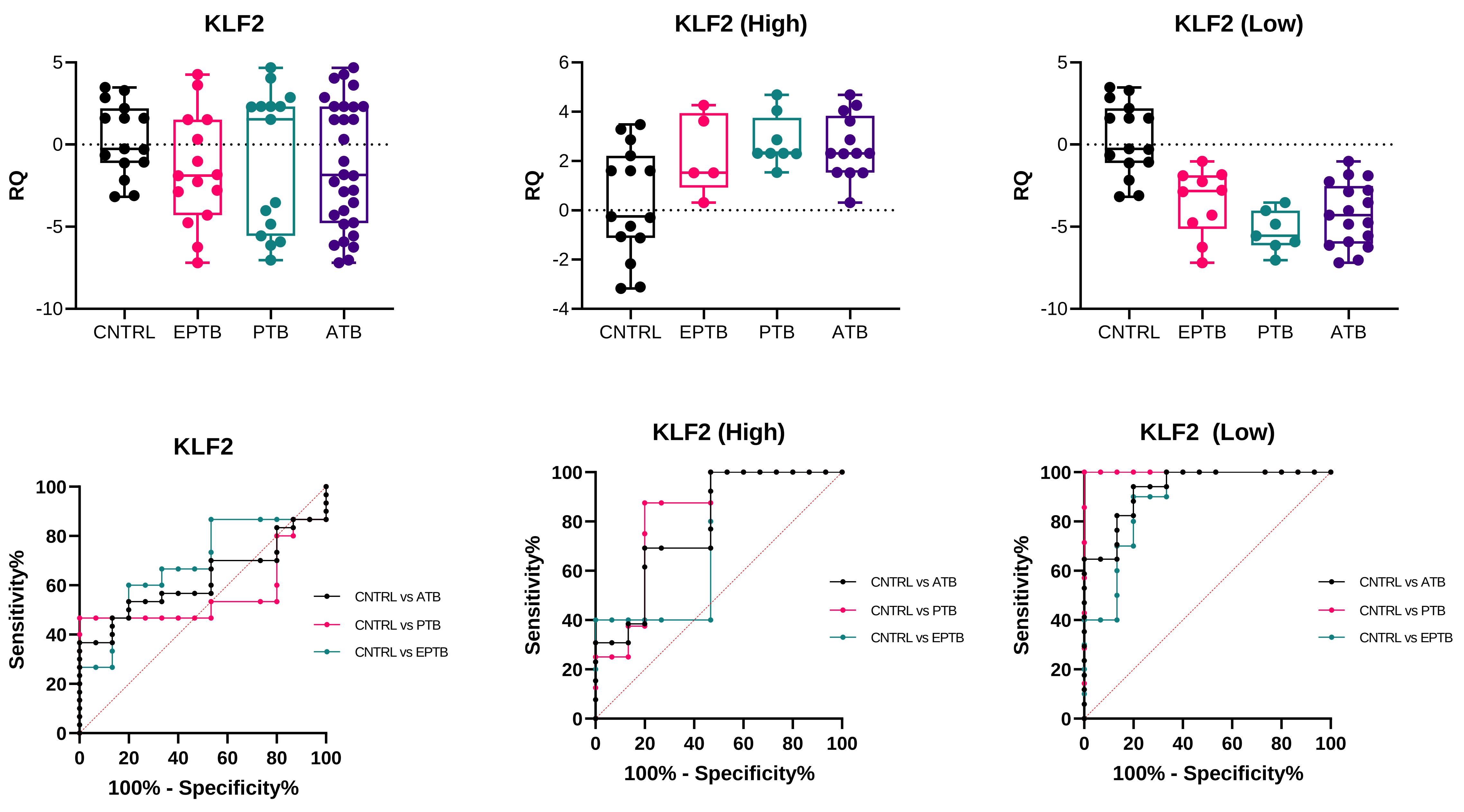
KLF2
RQ
5
0
-5
-10
CNTRL
EPTB
PTB
ATB
KLF2 (High)
6
4
2
0
-2
-4
KLF2 (Low)
Sensitivity%
100% - Specificity%
0
20
40
60
80
100
CNTRL vs ATB
CNTRL vs PTB
CNTRL vs EPTB
KLF2 (Low)

Figure S4.6

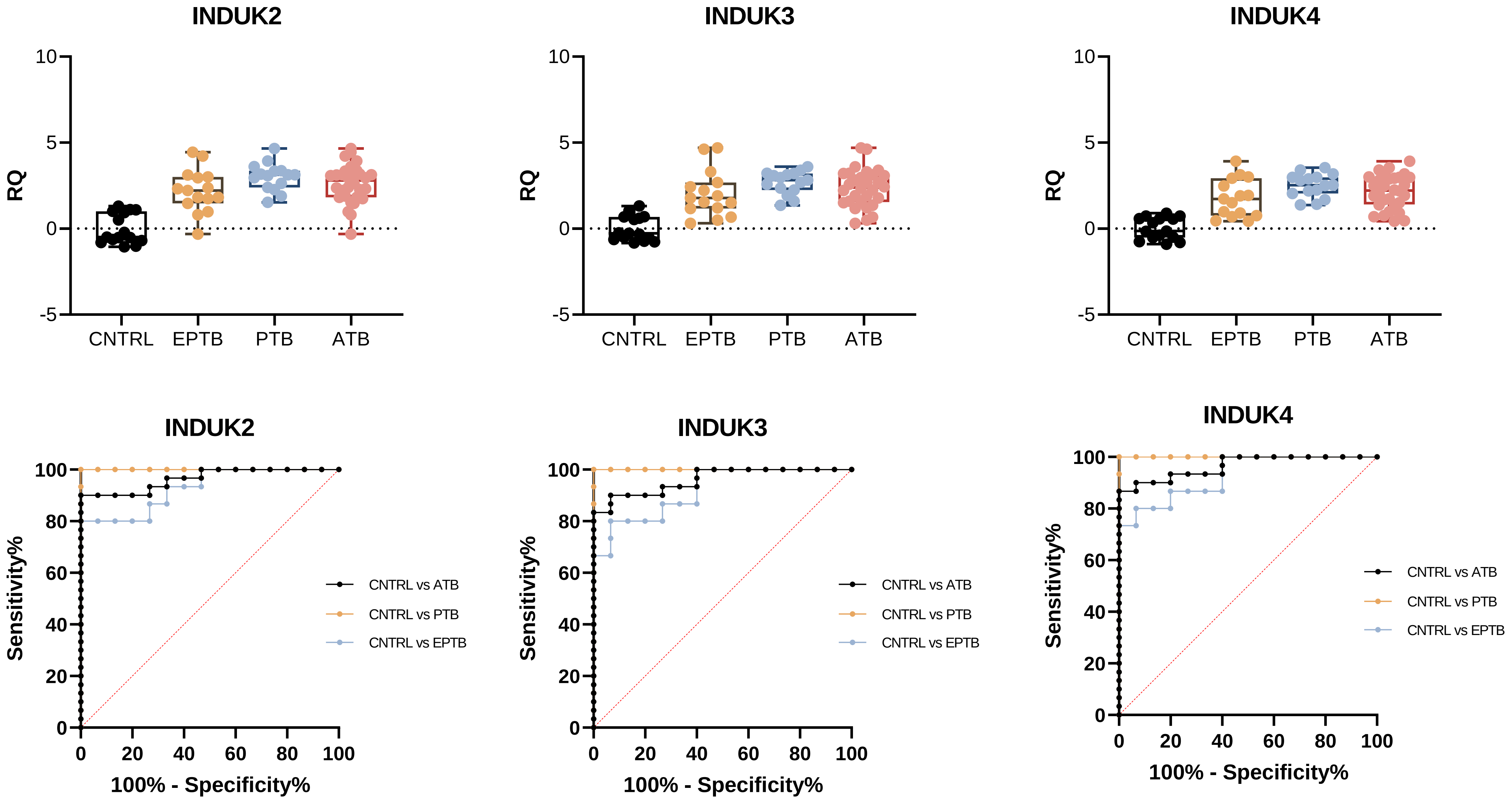

INDUK2
INDUK3
INDUK4
RQ
10
5
0
-5
CNTRL
EPTB
PTB
ATB
Sensitivity%
100% - Specificity%
0
20
40
60
80
100
CNTRL vs ATB
CNTRL vs PTB
CNTRL vs EPTB

Figure S4.7

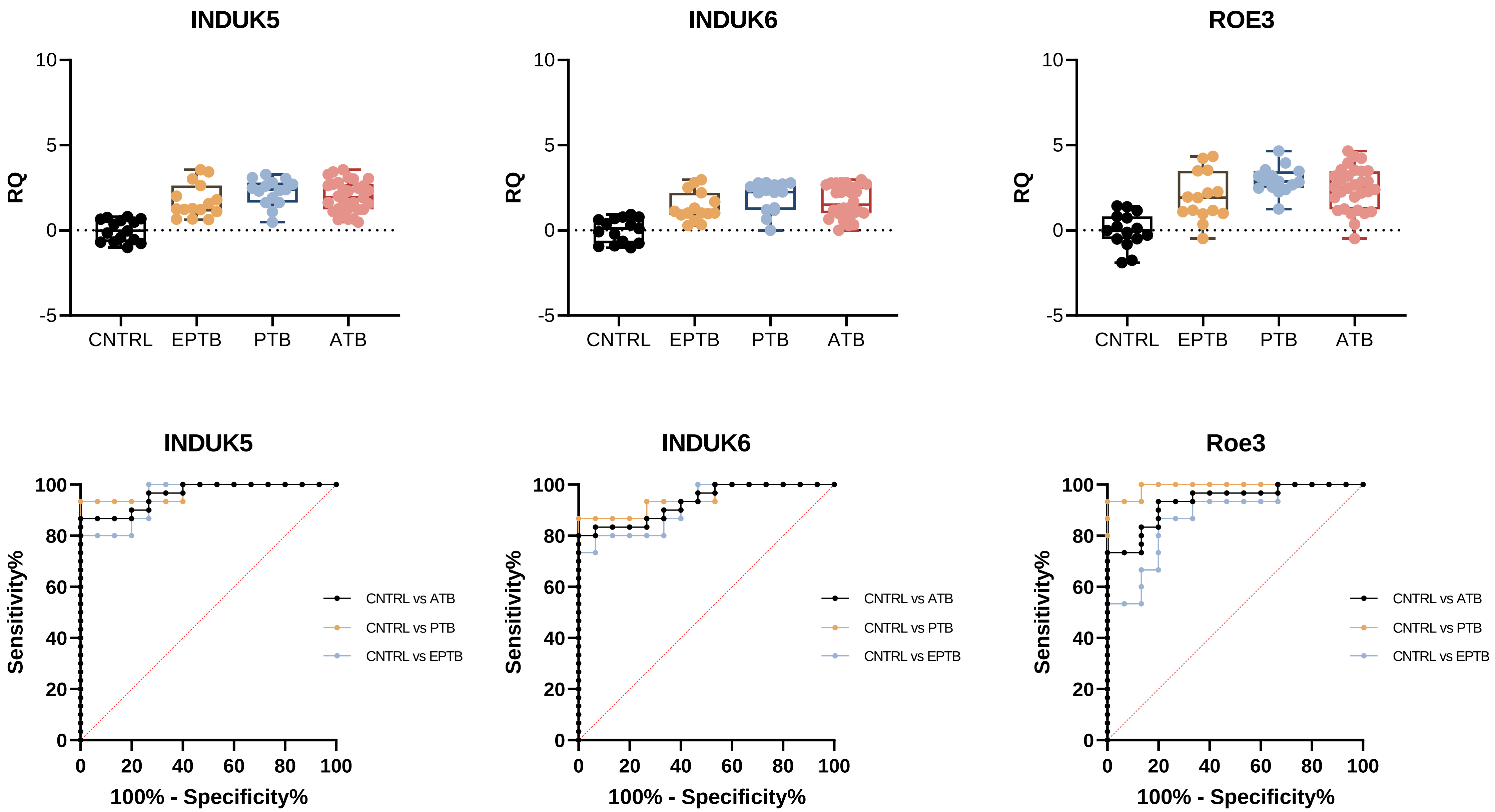
INDUK5
INDUK6
ROE3
RQ
10
5
0
-5
CNTRL
EPTB
PTB
ATB
INDUK5
INDUK6
Roe3
Sensitivity%
100% - Specificity%
0
20
40
60
80
100
CNTRL vs ATB
CNTRL vs PTB
CNTRL vs EPTB

Figure S4.8

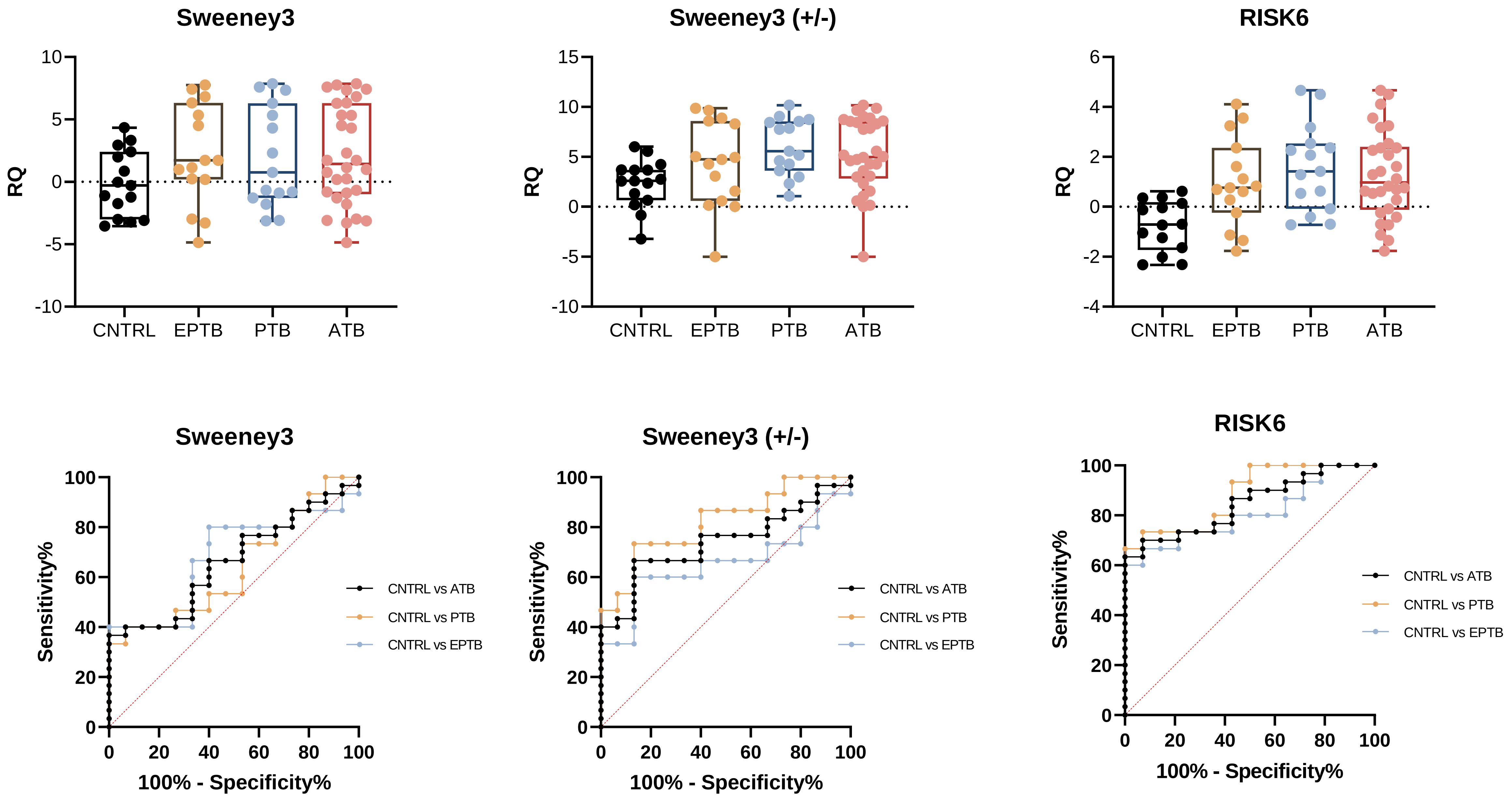
Sweeney3
Sweeney3 (+/-)
RISK6
RQ
CNTRL
EPTB
PTB
ATB
Sweeney3
Sweeney3 (+/-)
RISK6
Sensitivity%
100% - Specificity%
CNTRL vs ATB
CNTRL vs PTB
CNTRL vs EPTB

Figure S4.9

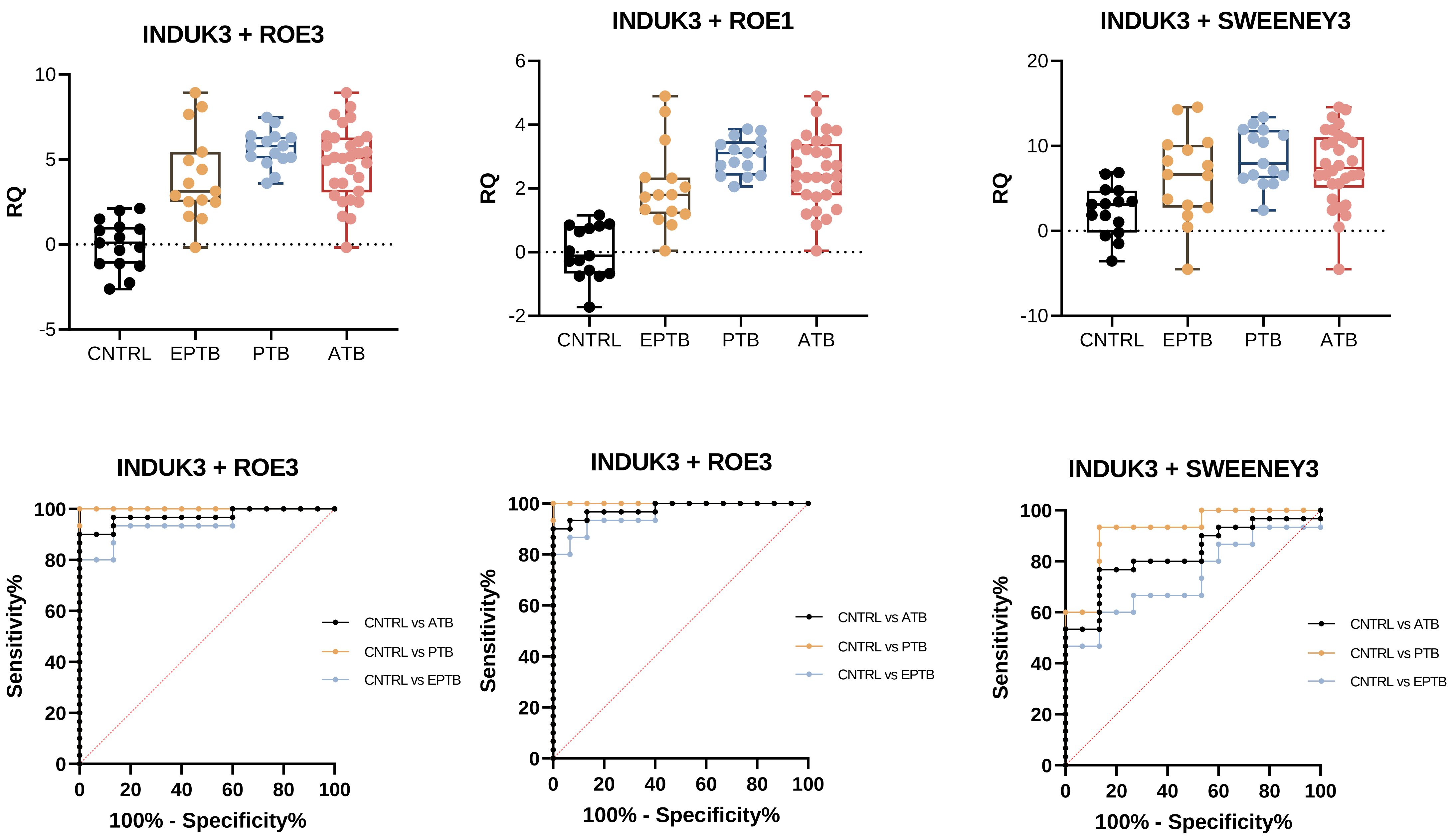

INDUK3 + ROE3
RQ
10
5
0
-5
CNTRL
EPTB
PTB
ATB
INDUK3 + ROE1
RQ
6
4
2
0
-2
CNTRL
EPTB
PTB
ATB
INDUK3 + SWEENEY3
RQ
20
10
0
-10
CNTRL
EPTB
PTB
ATB
INDUK3 + ROE3
Sensitivity%
100% - Specificity%
0
20
40
60
80
100
CNTRL vs ATB
CNTRL vs PTB
CNTRL vs EPTB
INDUK3 + ROE3
Sensitivity%
100% - Specificity%
0
20
40
60
80
100
CNTRL vs ATB
CNTRL vs PTB
CNTRL vs EPTB
INDUK3 + SWEENEY3
Sensitivity%
100% - Specificity%
0
20
40
60
80
100
CNTRL vs ATB
CNTRL vs PTB
CNTRL vs EPTB

Figure S4.10

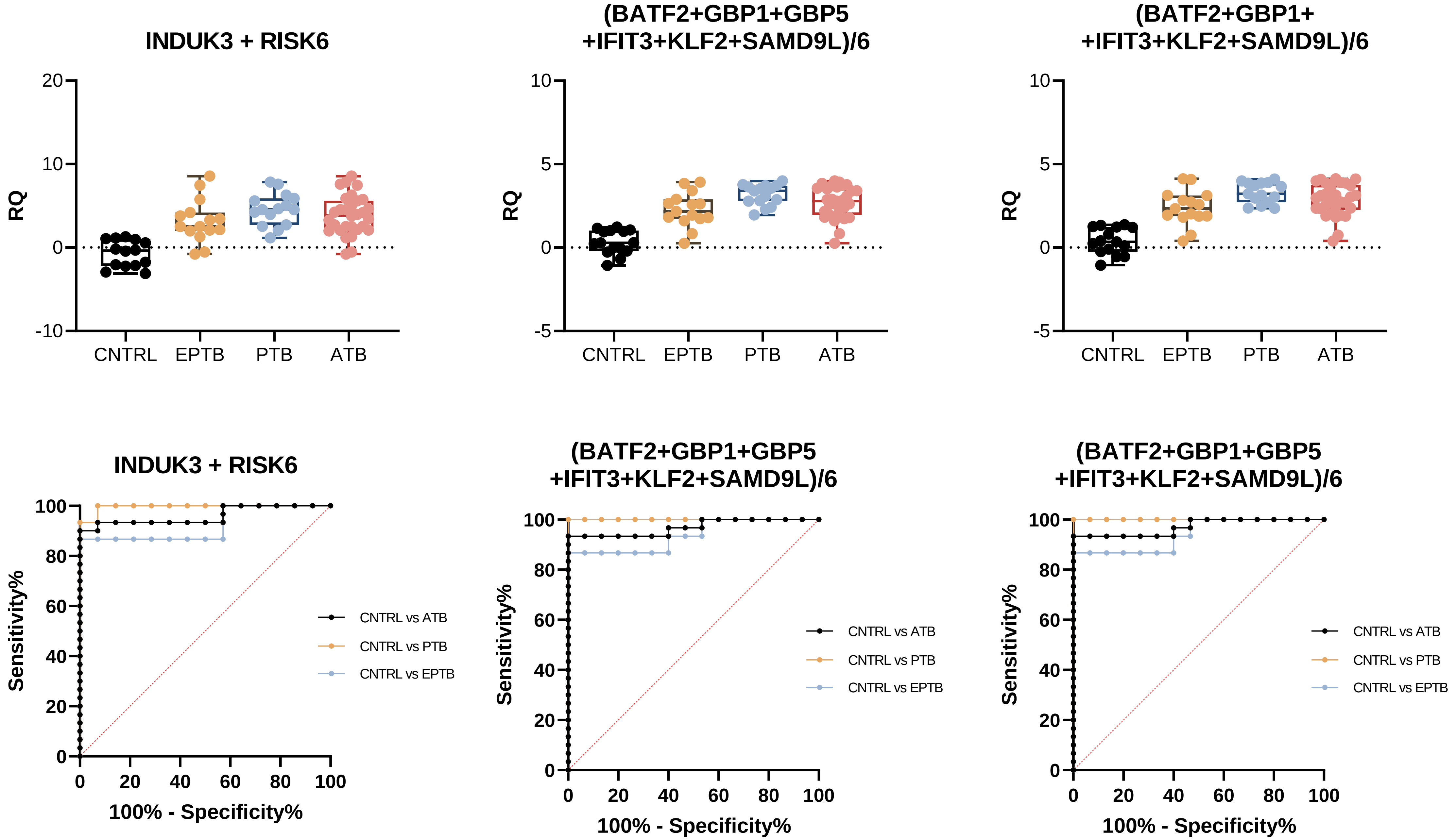

INDUK3 + RISK6
RQ
20
10
0
-10
CNTRL
EPTB
PTB
ATB
(BATF2+GBP1+GBP5
+IFIT3+KLF2+SAMD9L)/6
RQ
10
5
0
-5
CNTRL
EPTB
PTB
ATB
(BATF2+GBP1+
+IFIT3+KLF2+SAMD9L)/6
RQ
10
5
0
-5
CNTRL
EPTB
PTB
ATB
INDUK3 + RISK6
Sensitivity%
100% - Specificity%
CNTRL vs ATB
CNTRL vs PTB
CNTRL vs EPTB
(BATF2+GBP1+GBP5
+IFIT3+KLF2+SAMD9L)/6
Sensitivity%
100% - Specificity%
CNTRL vs ATB
CNTRL vs PTB
CNTRL vs EPTB
(BATF2+GBP1+GBP5
+IFIT3+KLF2+SAMD9L)/6
Sensitivity%
100% - Specificity%
CNTRL vs ATB
CNTRL vs PTB
CNTRL vs EPTB
