# Supplementary material for: Evaluation of host-immune biomarker signatures as multiplex qPCR diagnostic assays: a pilot study toward meeting WHO target product profiles for TB diagnosis in India
Source: Front Tuberc. 2026 Feb 16;3:1678039. doi: 10.3389/ftubr.2025.1678039 (PMC13494995; doi:10.3389/ftubr.2025.1678039)
Supplement: Supplementary file 2 [file Data_Sheet_2.docx]

**Figure S2.1**

(A)

(B)

**Table S2.1**

| **Biomarker** | **Week 1** | **Week 2** | **Week 4** | **Week 6** |
| --- | --- | --- | --- | --- |
| GBP1 | 0.6481 | 0.9043 | 1 | 1 |
| SNX10 | 0.6574 | 0.9043 | 0.9506 | 1 |
| GBP3/GBP1 | 0.5093 | 0.7994 | 0.9815 | 0.9815 |
| CALCOCO2 | 0.5833 | 0.8673 | 0.8673 | 0.9722 |
| SAMD9L | 0.5895 | 0.8611 | 0.8827 | 0.9414 |
| IFIT3 | 0.5031 | 0.8148 | 0.9969 | 0.9074 |
| SCARF1 | 0.6883 | 0.5556 | 0.5802 | 0.8735 |
| TUBGCP6 | 0.5154 | 0.5278 | 0.9043 | 0.8549 |
| KLF2 | 0.8272 | 0.9969 | 0.5864 | 0.7407 |
| SCARF1 | 0.5123 | 0.5494 | 0.5648 | 0.5988 |
| GBP5 | 0.5401 | 0.5679 | 0.5062 | 0.5741 |
| BATF2 | 0.5401 | 0.537 | 0.6821 | 0.5679 |
| DUSP3 | 0.5525 | 0.5247 | 0.6944 | 0.5432 |
| SERPING1 | 0.6481 | 0.7346 | 0.6451 | 0.5401 |
| SERPING1 | 0.7284 | 0.8272 | 0.7284 | 0.5093 |

**Figure S2.2**

**Figure S2.3**

(A)

(B)

**Figure S2.4**

(A)

(B)

**Figure S2.5**

**Figure S2.6**

(A)

(B)

**Figure S2.7**

(A)

(B)
